# One‐Pot Incorporation of Quantum Defects into Single‐Walled Carbon Nanotubes with Arylazo Sulfonates

**DOI:** 10.1002/anie.202521215

**Published:** 2025-12-23

**Authors:** Valeriia D. Andreeva, Justus T. Metternich, Chen Ma, Janus A.C. Wartmann, Sebastian Kruss

**Affiliations:** ^1^ Department of Chemistry and Biochemistry Ruhr University Bochum 44801 Bochum Germany; ^2^ Fraunhofer Institute for Microelectronic Circuits and Systems 47057 Duisburg Germany

**Keywords:** Arylazo sulfonates, Carbon nanotubes, Fluorescence, Photochemistry, Quantum defects

## Abstract

Single‐walled carbon nanotubes (SWCNTs) consist of a carbon monolayer and fluoresce in the near‐infrared (NIR, 800–2500 nm) region. Introduction of sp^3^ quantum defects (QDs) creates novel NIR emission features, which promise huge potential for (bio)photonics. However, so far defect chemistry based on diazonium salts is mainly limited to benzene derivatives. The reaction side products also quench SWCNT fluorescence and impair additional surface chemistry. Here, we introduce a photochemical strategy based on arylazo sulfonates to a) create more complex aromatic QDs and b) avoid side products that adsorb and quench. We show that this way coumarin and other QDs are incorporated, which increases overall NIR emission by more than 90% without purification. These materials can be non‐covalently modified with biocompatible polymers (polyethyleneglycoles, DNA) without a change in photophysical properties and we demonstrate mechanical (viscosity) and chemical (neurotransmitter dopamine) sensing. This one‐pot approach creates QDs in SWCNTs and provides access to advanced hybrid materials for photonic applications.

## Graphical abstract

We present a photochemical strategy to create complex aromatic quantum defects in carbon nanotubes using arylazo sulfonates. It provides access to brighter carbon nanotubes without purification and simpler biofunctionalization.

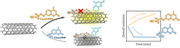

## Introduction

Solid state (nano)materials have versatile optoelectronic properties, but their synthesis often lacks the chemical precision that is known from molecular chemistry. Semiconducting single‐walled carbon nanotubes (SWCNTs) consist of sp^2^‐hybridized carbon monolayers and fluoresce in the beneficial near‐infrared (NIR) biological transparency window (800 – 1700 nm).^[^ ^1^, ^2^, ^3^ ^]^ SWCNTs can be chemically modified by cycloaddition reactions with subsequent rehybridization preserving the sp^2^ lattice.^[^ ^4^ ^]^ Covalent modification (i.e. change of hybridization from sp^2^ to sp^3^) is possible but typically destroys the NIR fluorescence.^[^ ^5^ ^]^

Interestingly, introducing a low number of covalent bonds (quantum defects, QDs) ^[^ ^6^, ^7^, ^8^, ^9^ ^]^ creates new red‐shifted emission features (E_11_^*^) with huge potential for quantum light sources, sensing or bioimaging. ^[^ ^10^, ^11^, ^12^, ^13^ ^]^ The shift depends on the electron‐withdrawing or ‐donating effects of the introduced group.^[^ ^6^ ^]^ Despite of the well‐described field of SWCNT chemistry, ^[^ ^5^, ^14^, ^15^, ^16^, ^17^, ^18^, ^19^, ^20^ ^]^ methods of sp^3^ QDs introduction, which also maintain SWCNT fluorescence are limited. So far reactions with diazonium salts,^[^ ^21^, ^22^ ^]^ oxygen doping with ozone under light irradiation,^[^ ^23^ ^]^ oxygen doping under ultraviolet (UV) light irradiation of 254 nm,^[^ ^24^ ^]^ oxidation,^[^ ^25^ ^]^ cycloaddition,^[^ ^26^ ^]^ and halogen‐assisted reactions^[^ ^27^, ^28^ ^]^ have been used.

Functionalization of SWCNTs with biologically relevant recognition moieties is limited by the necessity to work in aqueous buffer. An additional challenge is to avoid cross‐reactivity of linker groups with the SWCNT coating for colloidal stability.^[^ ^29^ ^]^ Known strategies include conjugation of peptides with SWCNTs via a defect‐free triazine linker strategy which does not generate a new emission feature,^[^ ^30^ ^]^ or through a maleimide‐aryldiazonium linker.^[^ ^31^, ^32^ ^]^ The triazine functionalization strategy consists of several steps that include purifications in between the chemical reactions. On the other hand, the diazonium chemistry is fast, green, has been demonstrated to work in aqueous solution, controlled by light irradiation and is compatible for the step‐wise functionalization e.g., via the maleimide functional moiety. Another possibility is to directly use quantum defects that are recognition units by themselves such as boronic acid containing QDs.^[^ ^33^, ^34^ ^]^

Previously, it was established that the reaction with aryl diazonium salts proceeds in at least two steps: ^[^ ^35^, ^36^, ^37^, ^38^ ^]^ (i) adsorption of the aryl diazonium salt on the surface of the SWCNT, which is characterised by quenching of the intrinsic bandgap emission (E_11_), and (ii) the sp^3^ QD introduction into the sp^2^ lattice of SWCNTs, characterized by an increasing E_11_^*^ red‐shifted emission feature. While the first step takes place immediately after the addition of the aryl diazonium salt, the second step has slower kinetics and can be followed by changes of the SWCNT emission.^[^ ^35^ ^]^ The resulting QDs containing SWCNTs are called colour centre carbon nanotubes (CCNT). Thus, this most‐common reaction pathway quenches the emission intensity of E_11_ peak of SWCNTs fluorescence at least for high diazonium salt concentrations and fast reaction times. Additionally, physisorbed hyrdophobic groups or reaction products make exchange to biocompatible polymers challenging.^[^ ^39^, ^40^, ^41^, ^42^, ^43^ ^]^

**Figure 1:**
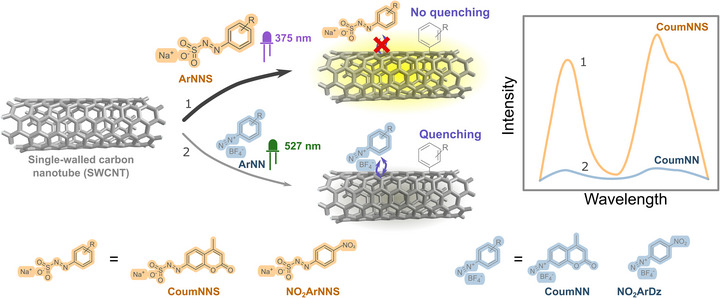
Alternative routes to quantum defects in carbon nanotubes. One‐pot sp^3^ quantum defect introduction into single‐walled carbon nanotubes (SWCNTs) via a photoreaction 1) with azosulfonates (**ArNNS**, **CoumNNS**, and orange). It avoids the physisorption of the hydrolysed side‐products that a) quench and b) impair non‐covalent modification, which is known from the conventional photoreaction 2) with diazonium salts (**ArNN**, **CoumNN**, and blue). It provides an alternative pathway to color center carbon nanotubes (CCNTs). Note that arylazo sulfonates can be easily obtained by adding sodium sulfite to a diazonium salt.

In this work we report the introduction of QDs with arylazo sulfonates into SWCNTs. We demonstrate the approach for standard diazonium salts and for the diazonium salt of a larger oxidation‐sensitive fluorophore (coumarin). We hypothesized that with a water‐soluble reaction species we overcome the typical adsorption of the reagent on the SWCNT surface, which causes strong quenching and is difficult to remove. We demonstrate coumarin‐based quantum defects and propose a mechanism for this novel one‐pot synthesis procedure (Figure 1). This approach opens the possibility to efficiently modify SWCNTs with QDs, which is relevant for applications in biosensing, bioimaging and quantum computing.

## Results and Discussion

**Figure 2:**
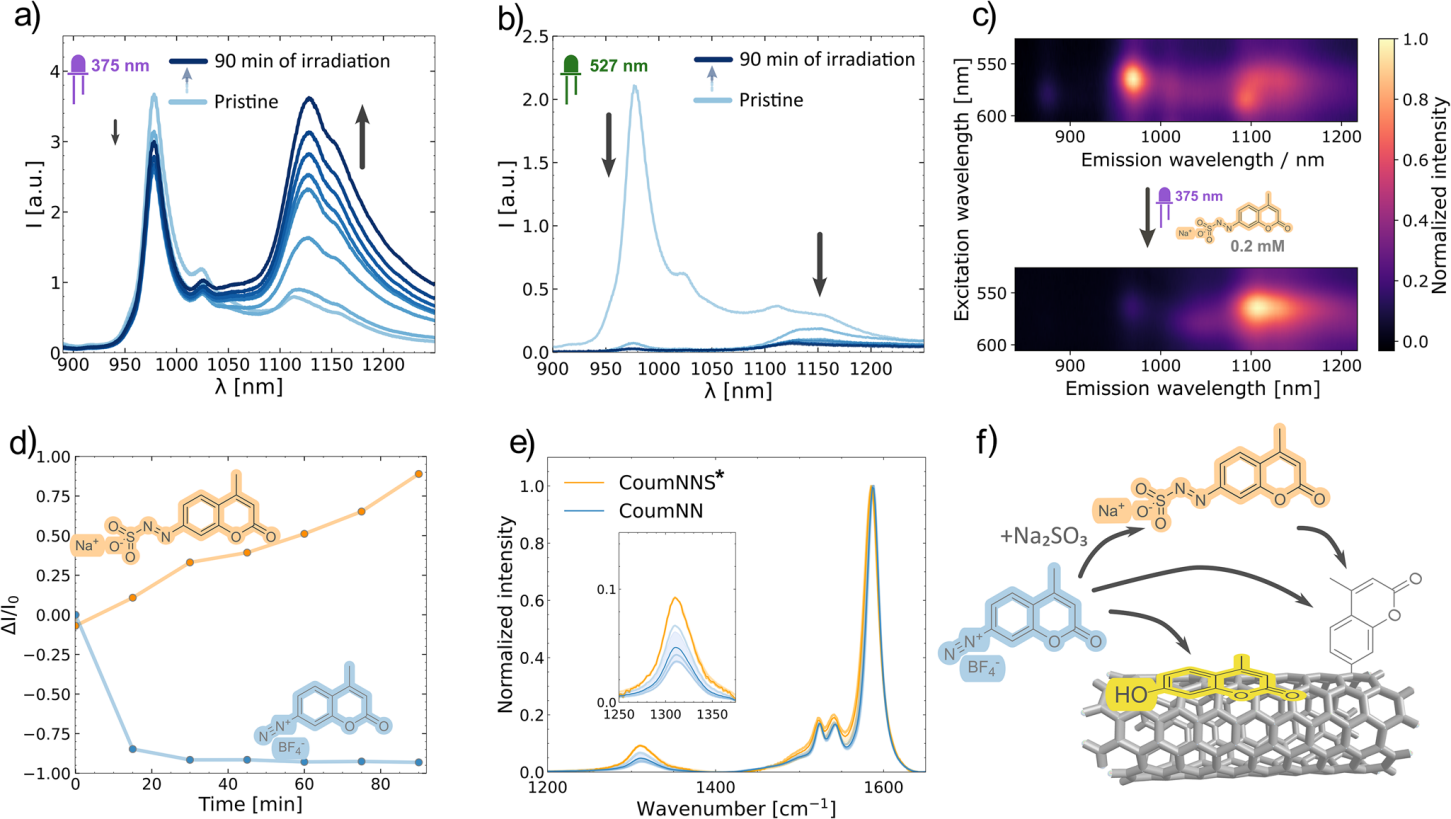
One‐pot quantum defect incorporation increases fluorescence without additional purification. a) Non‐normalized emission spectra of SDBS‐(6,5)‐SWCNTs over time during introduction of QDs by the photoreaction (375 nm) with **CoumNNS** (40 µM) without any purification. b) Non‐normalized emission spectra of SDBS‐(6,5)‐SWCNTs over time during introduction of QDs by the photoreaction (527 nm) with **CoumNN** (50 µM) without any purification. c) 2D excitation‐emission spectra of SDBS‐(6,5)‐SWCNTs mixed with 0.2 mM of **CoumNNS** right after mixture and after 90 min of irradiation with 375 nm light. d) Overall emission intensity during the reaction of SDBS‐(6,5)‐SWCNTs with **CoumNNS** under UV light (375 nm, yellow line) and **CoumNN** under green light (527 nm, blue line). Here *I* is the integral over the emission spectrum [900 – 1210 nm], *I_0_* is the emission of pristine SDBS‐(6,5)‐SWCNTs. e) Raman spectra of SDBS‐(6,5)‐CCNTs prepared by the photoreaction with 50 µM of **CoumNNS** (yellow) and **CoumNN** (blue) normalized to the G peak (each is depicted as the bold mean line ± shaded SD, *n* = 3, Raman *λ*_exc_ = 532 nm). f) Proposed mechanism. **CoumNN** in contrast to **CoumNNS** hydrolyses into the more hydrophobic **CoumOH**, which most likely physisorbs and quenches the SWCNT fluorescence.

We hypothesized that hydrolysis of diazonium salts into more hydrophobic compounds causes the problematic physisorption of non‐wanted side products onto the SWCNT surface. Thus, we aimed to make the diazonium salt more hydrophilic and at the same time more stable. As the effect should be biggest for larger aromatic compounds we decided to explore such compounds first. To investigate how the reagent solubility influences the final CCNT's emission intensity, we tested the coumarin sp^3^ defect introduction with freshly prepared sodium 2‐(4‐methylcoumarin)diazene‐1‐sulfonate (**CoumNNS**) fully soluble in water. **CoumNNS** was prepared from the 4‐methylcoumarin‐7‐diazonium salt (**CoumNN**). **CoumNN** itself was prepared by a previously reported procedure (Figure S1).^[^ ^31^ ^]^ The diazonium salt was then dissolved in water solution of Na_2_SO_3_ to generate **CoumNNS** (Figures S2–S5). When we added freshly prepared **CoumNNS** to the dispersion of sodium dodecyl benzene sulfonate – coated (6,5)‐enriched SWCNTs (**SDBS‐(6,5)‐SWCNT**) and irradiated the samples with 375 nm UV LED the E_11_^*^ emission strongly increased (Figure 2a,c). It led to 89% more NIR emission of SDBS‐(6,5)‐CCNTs compared to pristine SDBS‐(6,5)‐SWCNTs. Overall, the E_11_ maximum emission wavelength (around 1000 nm) of the SDBS‐(6,5)‐CCNTs prepared by the conventional approach was smaller compared to the SDBS‐(6,5)‐CCNTs functionalized with **CoumNNS** leading to the drastic difference in overall emission of the functionalized SDBS‐(6,5)–CCNTs (Δ*I*/*I*_0_ = 0.89 vs ‐0.93, respectively, Figure 2d). In comparison to the non‐purified **CoumNN** sample (Figure 2c) this approach leads to 2685% brighter samples.

We observed the opposite effect on the overall fluorescence emission for the established photoreaction protocol of aryl diazonium salts such as **CoumNN**.^[^ ^31^, ^35^ ^]^ The E_11_ emission decreased much more than in the experiment with **CoumNNS**. Moreover, the relative increase in E_11_^*^ emission did not exceed the initial emission intensity at the same wavelength (Figure 2c). Different concentrations of **CoumNN** were added and every time quenching was observed (Figure S11). It can be attributed to the first step of the reaction, which has been described as adsorption of the aromatic diazonium salt^[^ ^35^, ^38^ ^]^ on the surface resulting in decrease of the E_11_ emission.

Therefore, we hypothesized that more hydrophobic reagents cause more quenching, which outcompetes the sp^3^ defect related increase of emission and causes an overall fluorescence decrease. Diazonium salts most probably will convert into the hydrophobic hydroxy derivatives (Figure S6, **CoumOH**).To show the difference in number of coumarin‐based QDs, we prepared samples by both reactions in such manner that the resulting emission spectra of the SDBS‐(6,5)‐CCNTs at the same concentration were almost identical (Figure S12). For that we made the QD introduction reactions with premixed CoumNN with Na_2_SO_3_ in 1:1 molar ratio in water (CoumNNS^*^), and **CoumNN**. The amount of introduced sp^3^ QDs in the samples was analysed with Raman spectroscopy. We observed the higher D/G ratio in the CoumNNS^*^ samples than in the samples after the reaction with **CoumNN** (0.15 and 0.09, respectively; Figure 2e), which corresponds to a higher defect density.^[^ ^44^ ^]^ The lower amount of introduced sp^3^ QDs per sample by the conventional method at the same emission intensity in the NIR suggests emission quenching by the physisorption of **CoumNN** molecules on the SWCNT. Based on the results of NIR emission, NMR and Raman studies we could validate our hypothesis (Figure 2f).

Noteworthy, when the QD introduction was performed with **CoumNNS** and **CoumNN** at the same reactant concentrations (40 µM), the conventional sample showed higher defect densities. However, the emission intensities are drastically higher for the **CoumNNS** case (Figure S13). If one aims for higher defect densities one can adjust the reactant concentration accordingly but the main advantage of much higher brightness remains.

The photoarylation reaction via photolysis of arylazo sulfones is well‐described in organic solvents,^[^ ^45^, ^46^ ^]^ and recently the photoreaction of arylazo sulfonates in water and water‐acetonitrile or alcohol mixtures was explored.^[^ ^47^ ^]^ The reaction pathway of arylazo sulfones in organic media was established to be dependent on the irradiation wavelength (UV or visible) because different excited states were populated. Thus, under the UV irradiation of the azosulfones in organic media, the singlet excited state ^1^ππ^*^ is formed which converts into the triplet excited state ^3^ππ^*^ leading to the –N─S─ bond heterolysis, followed by the N_2_ leave and formation of the reactive triplet cation ^3^Ar^+^. The blue light irradiation of the arylazo sulfones in otherwise the same conditions leads to the ^1^nπ^*^ excited state, followed by homolysis of the –N─S─ bond and formation of the aryl radical Ar·. Radical scavengers in this case lowered the yield of the reaction under visible light irradiation.^[^ ^45^, ^46^, ^48^ ^]^ On the other hand, formation of the aryl radical from the arylazo sulfonates in neat water solutions is less efficient and the influence of the irradiation wavelength on the reaction is negligible.^[^ ^47^ ^]^

**Figure 3:**
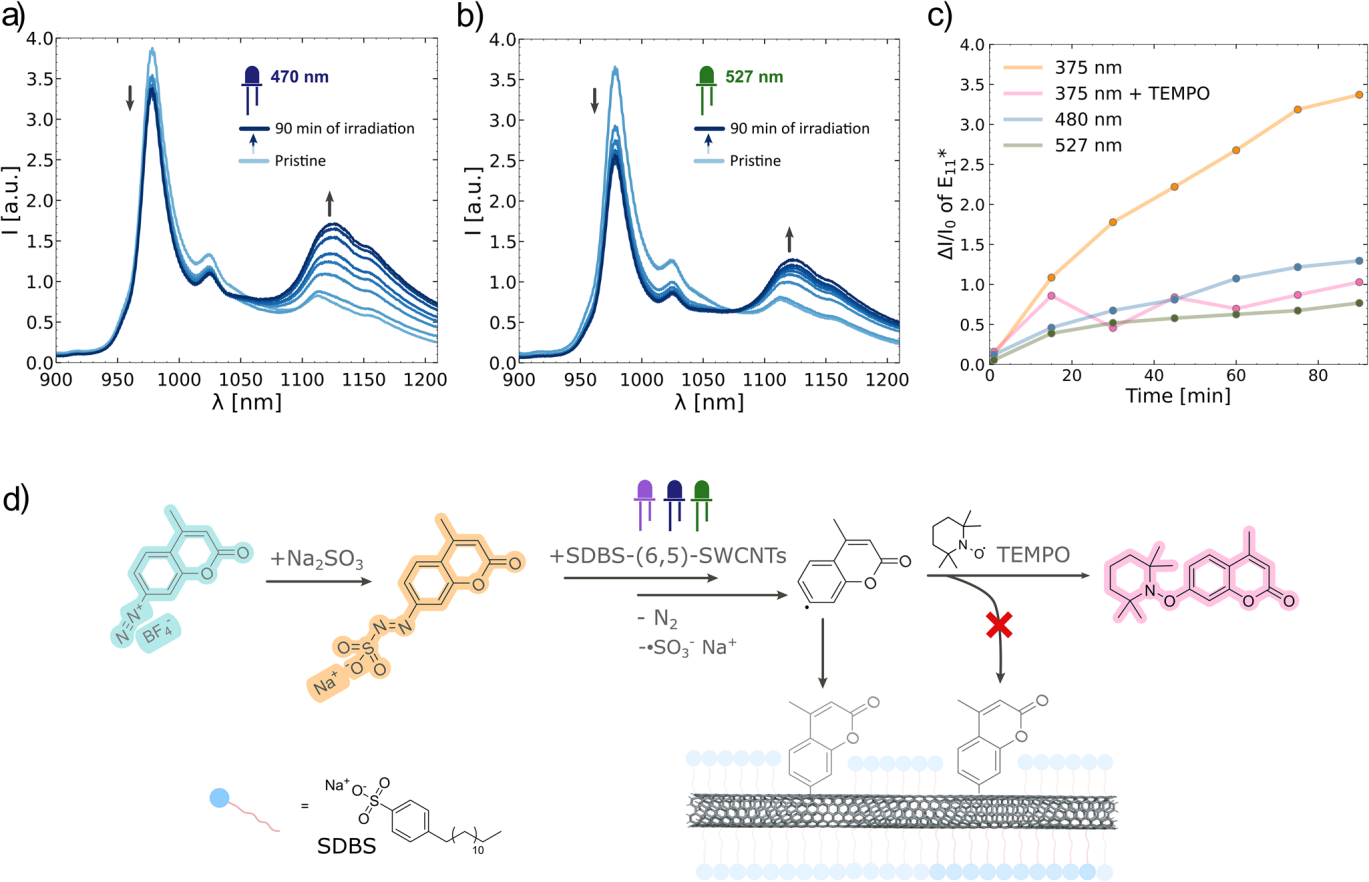
Quantum defect incorporation via arylazo sulfonates photolysis proceeds via a wavelength‐dependent radical mechanism. a) Fluorescence spectra of the photoreaction of SDBS‐(6,5)‐SWCNTs with 40 µM of **CoumNNS** under 470 nm blue light over time. b) Fluorescence spectra of photoreaction of SDBS‐(6,5)‐SWCNTs with 40 µM of **CoumNNS** under 561 nm green light over time. c) Emission intensity changes of E_11_^*^ ( = QD incorporation) depends on excitation wavelength and can be inhibited by radical scavengers. d) Proposed mechanism of SWCNT functionalization with **CoumNNS**.

Knowing these patterns in the photoarylation with arylazo sulfones and arylazo sulfonates we investigated the QD introduction reaction under different irradiation light wavelength from UV (375 nm, Figure 2b) to blue (470 nm), and green (561 nm), (Figures 3a,b, and S14, S15). The E_11_^*^ intensity over time (Figure 3c) showed strong differences, which suggests that a radical mechanism is involved. The radical nature of the reaction with **CoumNNS** under the mild UV light irradiation was further confirmed by repeating the reaction in the presence of 3 equivalents of the radical scavenger 2,2,6,6‐Tetramethylpiperidinyloxyl (**TEMPO**), added after 15 min of irradiation (Figures 3c and S16). The E_11_^*^ intensity did not further increase after the addition of **TEMPO**. A similar tendency was observed when the samples were irradiated with green light (Figure S17). These experiments show that QD introduction occurs via a radical reaction under both UV and visible light irradiation. However, under visible light irradiation, the formation of the coumaryl radical is hindered but not entirely abolished. Nevertheless, a similar E_11_^*^/E_11_ ratio can be achieved with higher concentrations of the arylazo sulfonates under visible light irradiation. Formed this way coumaryl radical attacks the sp^2^ lattice of SWCNTs forming a covalent bond. We therefore propose that after the initiation of the radical reaction, it follows the previously described path (Figure 3d).^[^ ^6^, ^49^ ^]^ The formed E_11_^*^ peak consists of two emission maxima: 1130 and 1160 nm (Figures 2a, 5a, and S22). It is most likely related to the formation of a second QD. After the formation of a bond with coumarin the adjacent carbon atom with a radical character could lead to a second sp^3^ QD with different geometry (ortho‐, para‐) relative to the coumarin QD.^[^ ^50^, ^51^ ^]^

Next, we investigated the SWCNT functionalization via in situ formed **CoumNNS**. Formation of the **CoumNNS** from **CoumNN** occurs much faster than the introduction of sp^3^ QD into the SDBS‐(6,5)‐SWCNTs. Thus, we performed the reaction with in situ formed **CoumNNS** under green light irradiation. In these experiments, Na_2_SO_3_ was added to the SDBS‐(6,5)‐SWCNT samples with varying concentrations (5, 2.5, and 1 mM, Figure S18). The **CoumNN** solution was added to the samples and it was irradiated with green light for various durations. Under these reaction conditions we observed almost no E_11_ emission quenching and increasing E_11_^*^ emission resulted in increase of the overall emission of the functionalized SDBS‐(6,5)‐CCNTs compared to pristine sample containing 5 mM of Na_2_SO_3_. However, the reaction progression (E_11_^*^/E_11_ ratio) slowed down with increase of the Na_2_SO_3_ concentration. No E_11_^*^ peak formation was observed in the control experiment (Figure S19). These results show that in situ formation and reaction of arylazo sulfonates with SWCNTs is possible. When comparing the 2D spectra of **CoumNN** and **CoumNNS** we observed no difference in the E_11_* emission pattern (Figure S22b, c) upon modification in the presence or absence of the Na_2_SO_3_, which would be expected for C─C versus C─S bond based defects. Therefore, we propose that the ·SO_3_^−^Na^+^ radical formed upon light irradiation did not form the sp^3^ QD. We assume that in this reaction conditions charged ·SO_3_^−^Na^+^ radical species prefer less hydrophobic environment than the SWCNTs’ surface and escape the reaction site before C─S bond formation. It is important to mention that, Na_2_SO_3_ is known as a ground‐state oxygen scavenger and, at a concentration of 5 mM, imitates oxygen‐free conditions.

**Figure 4:**
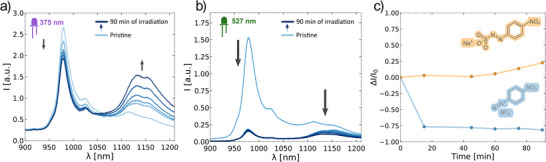
Extension of QD introduction to arylazo sulfonates. a) Non‐normalized emission spectra of SDBS‐(6,5)‐SWCNTs over time during introduction of QD by photoreaction (375 nm) with **NO_2_ArNNS** (40 µM) without any purification. b) Non‐normalized emission spectra of SDBS‐(6,5)‐SWCNTs over time during introduction of QD by photoreaction (527 nm) with **NO_2_ArDz** (10 µM) without any purification according to reported procedures.^[^ ^35^ ^]^ c) Overall emission intensity during the reaction of SDBS‐(6,5)‐SWCNTs with **NO_2_ArNNS** under UV light (375 nm, yellow line) and **NO_2_ArDz** under green light (527 nm, blue line. Here *I* is the integral over the emission spectrum [900 – 1210 nm], *I_0_* is the emission of pristine (SDBS‐(6,5)‐SWCNTs).

To demonstrate the transferability of the Na_2_SO_3_ – assisted reaction, and to compare it to the conventional method, we performed the sp^3^ introduction reaction with sodium 2‐(*p*‐nitro‐aryl)diazene‐1‐sulfonate (**NO_2_ArNNS,** Figures 4a, S7–S9) under the conditions for **CoumNNS** and compared it to the reaction with p‐nitro‐aryl diazonium salt (**NO_2_ArDz,** Figure 4b) made under the previously reported conditions.^[^ ^35^ ^]^ For this purpose, we added 40 µM of the **NO_2_ArNNS** to the SDBS‐(6,5)‐SWCNTs in the first case and 10 µM of the **NO_2_ArDz** for the reproduction of the conventional reaction. The reaction with **NO_2_ArNNS** was irradiated (375 nm) and the reaction with **NO_2_ArDz** was performed under 527 nm light. In these reactions we observed a similar tendency to the reactions with coumarin derivatives. The overall emission intensity dropped drastically in the conventional reaction with **NO_2_ArDz**, while the brightness of the SDBS‐(6,5)‐CCNTs prepared via the arylazo sulfonate path did not decrease at all and increased after 45 min of the reaction (Figure 4c). This reaction could also be performed in situ similarly to the reaction with **CoumNNS** (Figure S20).

Overall, these results support our hypothesis that the decrease in overall emission intensity during the sp^3^ QD introduction with diazonium salts is caused by the adsorption of the reagent on the surface of the SWCNT. When the adsorption is eliminated by creating a more hydrophilic reagent, the overall emission does not decrease anymore. We anticipate that with the use of the reagents possessing distinct, red‐shifted absorption wavelengths, the radical formation under mild UV light (375 nm) will be even more efficient and the overall emission will increase.

As we have developed an adsorption‐free sp^3^ introduction method, we hypothesized that the (6,5)‐CCNTs obtained this way will preserve the E_11_^*^/E_11_ ratio after surfactant exchange as the E_11_ emission is not quenched in the first place. Indeed, the exchange of the SDBS surfactant to 1,2‐dimyristoyl‐sn‐glycero‐3‐phosphoethanolamine‐N‐[methoxy(polyethylene glycol)‐5000] (ammonium salt) (**PEG‐PL**) resulted in the (6,5)‐PEG‐PL‐CCNTs with the same E_11_^*^/E_11_ ratio but an expected red‐shifted emission as result of change in polymer wrapping (Figure S21).^[^ ^52^ ^]^ The relative increase of emission intensity around 1040 nm might be explained by the PEG‐PL preference for other chiralities present in the CoMoCAT (6,5)‐SWCNTs sample or certain quantum defect geometries (Figure S22). Contrary the exchange to ssDNA was sequence‐dependent and the exchange mechanism appears to be more complex (Figure S23). Based on the preservation of the E_11_^*^/E_11_ ratio after the surfactant exchange and a high number of QDs with brighter overall emission, we conclude that the emission pattern obtained right after the QD introduction is not affected by physisorption and can be used directly for analysis without additional purification after the reaction. It is therefore more straightforward compared to the (6,5)‐SWCNTs chirality enrichment using polyfluorene‐based polymer sorting method.^[^ ^53^ ^]^

**Figure 5:**
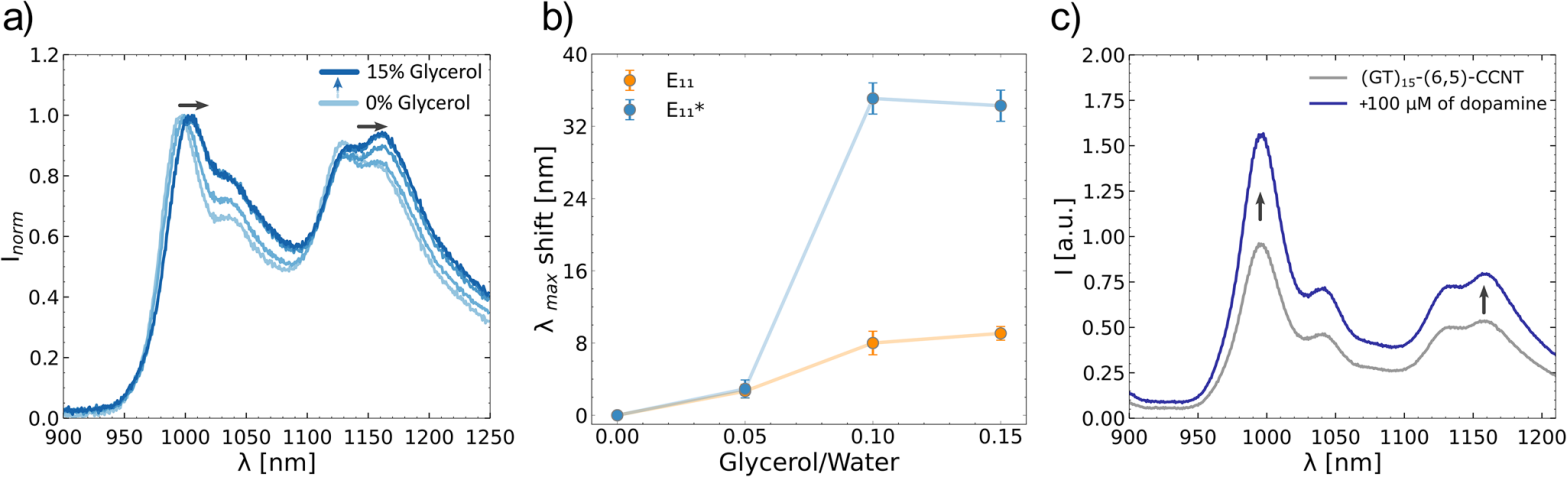
Mechanical and chemical sensing with coumarin quantum defect modified carbon nanotubes. a) Viscosity‐dependent fluorescence changes of PEG‐PL‐(6,5)‐CCNTs. b) E_11_ (yellow) and E_11_^*^ (blue) fluorescence wavelength shift depends on glycerol/water composition and thus viscosity (mean ± SE, *n* = 3). c) Dopamine (100 µM) increases overall fluorescence of (GT)_15_‐(6,5)‐CCNTs sample in PBS.

The coumarin‐based sp^3^ QD is asymmetric, and its rotation might influence the emission of PEG‐PL‐(6,5)‐CCNT. We hypothesized that with the increase of viscosity, the rotation along the C─C axis of the covalent bond between coumarin and the SWCNT is affected.^[^ ^54^ ^]^ Indeed, increasing volume fractions of glycerol (up to 15% glycerol/water volume ratio), shifted the fluorescence emission (Figure 5a,b). This mechanism is therefore different from a previously demonstrated ratiometric viscosity probe based on a quantum defect. ^[^ ^54^ ^]^ Our hypothesis is that the rotation of the coumarin moiety changes the local energy states geometry in the SWCNT and thus the emission wavelength. This effect could be different in magnitude for the two types of QDs with emission maxima at 1130 and 1160 nm, leading to a change of emission's maximum wavelength.

To explore the influence of coumarin‐based defects on the ssDNA‐based corona phase, we first exchanged the surfactant to (GT)_15_‐ssDNA and then added 100 µM of dopamine. (GT)_15_‐ssDNA functionalization is known to make SWCNTs highly sensitive sensors for catecholamine neurotransmitters such as dopamine.^[^ ^55^, ^56^ ^]^ Interestingly, the overall emission increased, while the E_11_^*^ emission increased less than the E_11_ emission (Figures 5c and S24). This observation may be explained by the increased exposure of the coumarin defect to the water environment as the result of corona phase – based dopamine recognition. Previously, the tendency was reported that with the increase of amount of the other QDs sensitivity of the nanosensor to catecholamines decreases.^[^ ^43^ ^]^ In this case (GT)_10_ was covalently attached to the CCNTs via the guanine‐based QDs and the QD was NO_2_Ar‐based. As the coumarin‐based defect of the (GT)_15_‐(6,5)‐CCNT is larger it could have a bigger impact on sensing. Our results show that neurotransmitter sensors based on DNA/SWCNTs can be modified QDs with the potential to make them brighter without losing their sensitivity and chemical selectivity (Figure S25).

Overall, the presented one‐pot approach comes with the great advantage that physisorption of diazonium salt reaction products do not impact the additional surface chemistry that is always required to guarantee colloidal stability in aqueous solution.^[^ ^43^ ^]^ It was one of the reasons why exchange to the most used biopolymer DNA has been difficult and yields were very low thus preventing the use of QDs as an additional chemical dimension in the design of nanosensors. Another convenient advantage is that the quantum yield (Figure 2a) is roughly 100% higher compared to the starting material and 2685% relative to the standard procedure. Of course, purification e.g. via dialysis is an option that decreases this advantage but novel quantum defect chemistry is explored using hundreds of reactants and conditions ^[^ ^57^ ^]^ and high throughput characterization is important. Our results also explain some discrepancies in the reported quantum yields or fluorescence increases of SWCNTs modified with diazonium salts.^[^ ^28^, ^51^ ^]^ It clearly shows that with no or reduced physisorption of reaction side products the overall brightness is higher. For applications in biosensing this much easier exchange from surfactants to biopolymers is providing access to higher yields and better signal/noise spectra.

## Conclusion

In conclusion, we have developed an efficient method of sp^3^ QD introduction that prevents fluorescence quenching of carbon nanotubes and physisorption of reaction products. We showcase this method for a larger coumarin‐based quantum defects but demonstrate in general that this arylazo sulfonate method provides significant advantages in terms of purification and biofunctionalization. An important insight is that the quantum yield increases even without purification. The chemical approach thus provides brighter NIR fluorescent SWCNTs and access to an additional chemical dimension (quantum defects) for applications in imaging, biosensing and quantum sensing.

## Supporting Information

The authors have cited additional references within the Supporting Information. ^[^ ^41^, ^58^, ^59^, ^60^, ^61^ ^]^

## Conflict of Interests

The authors declare no conflict of interest.

## Supporting information



Supporting Information

## Data Availability

The data that support the findings of this study are available from the corresponding author upon reasonable request.
