# Supplementary material for: One‐Pot Incorporation of Quantum Defects into Single‐Walled Carbon Nanotubes with Arylazo Sulfonates
Source: Angew Chem Int Ed Engl. 2025 Dec 23;65(6):e21215. doi: 10.1002/anie.202521215 (PMC12865254; doi:10.1002/anie.202521215)
Supplement: Supplementary file 1 — Supporting Information [file ANIE-65-e21215-s001.pdf]

Supporting Information

One-pot incorporation of quantum defects into single-walled carbon nanotubes with arylazo sulfonates

Valeriia D. Andreeva,[a] Justus T. Metternich,[a],[b] Chen Ma,[a] Janus A.C. Wartmann, [a] Sebastian Kruss*[a],[b]

[a] Dr. V.D. Andreeva, Dr. J.T. Metternich, Dr. C. Ma, J.A.C. Wartmann, Prof. S. Kruss
Department of Chemistry and Biochemistry
Ruhr University Bochum
44801, Bochum, Germany
E-mail: sebastian.kruss@rub.de

[b] Dr. J.T. Metternich, Prof. S. Kruss
Fraunhofer Institute for Microelectronic Circuits and Systems
47057, Duisburg, Germany

Contents

Experimental Procedures 2

1. Materials and general methods 2
2. Synthesis of 7-diazo-4-methylcoumarin tetrafluoroborate (CoumNN) 2
3. Synthesis of arylazosulfonates 3
4. (6,5) - SWCNT sample preparation 4
5. sp^3 quantum defect introduction with diazonium salts 4
6. Na_2SO_3 - assisted sp^3 quantum defect introduction 5
7. Mechanistic experiments 5
8. MeOH and ^{i}PrOH - assisted surfactant exchange to ss-DNA and PEG-PL 6
9. Dopamine, epinephrine and riboflavin response studies 6

Supplementary figures 7

Experimental Procedures

1. Materials and general methods

(6,5) – enriched single-walled carbon nanotubes ((6,5) – SWCNTs, Chasm Advanced Materials, Signis® SG65i, CoMoCAT™, ≥ 95% carbon basis (≥ 95% as carbon nanotubes, ≈ 41% as (6,5)-SWCNTs), 0.78 nm average diameter), sodium dodecylbenzenesulfonate (SDBS), 1,2-dimyristoyl-sn-glycero-3-phosphoethanolamine-N-[methoxy(polyethylene glycol)-5000] (ammonium salt) (PEG-PL; Avanti Research™) and single-stranded DNA sequences $(GT)_{10}$ and $(GT)_{15}$ were purchased from Sigma Aldrich GmbH. Reagents and solvents were purchased from Sigma Aldrich GmbH, Fisher Scientific GmbH, Chemspace LLC and Carl Roth GmbH + Co. KG. Solvents were used directly if not stated otherwise. All aqueous solutions were prepared in MilliQ® (MQ) water. NMR spectra were recorded on a Brucker AV-300 spectrometer at 298 K. Recorded spectra were analyzed with the MestReNova software.

Mounted LED M375L3 from Thorlabs Inc. at maximum power was used as the light source for reactions under 375 nm LED light. 4-wavelength high-power LED LED4D234 operated by the DC4100 driver (both from Thorlabs, Inc.) at the maximum power was used as the light source for reactions under 470 nm LED light. Lumidox® LED Array equipped with the LUMCON controller and LUM96G-GREEN (all Analytical Sales and Services, Inc.) at 25 mA was used as the light source for reactions under 527 nm green LED light.

Absorption spectra were recorded with a Jasco V-770 UV–visible/NIR Spectrophotometer in a polystyrene cuvette in the wavelength range of 400-1350 nm, if not stated otherwise.

NIR-emission spectra were recorded on a custom-built setup, described elsewhere.[1] In short, a Quantum gem561 diode solid state laser (561 nm) was used for excitation using an Olympus LCPLN-IR objective (20x, NA = 0.45) on a Olympus IX73 microscope connected to a Andor Technology Shamrock 193i spectrograph, equipped with an Andor InGaAs 491 detector. Spectra were smoothed using a gliding average to correct for odd-even effects of the detector. Recorded spectra of absorption and emission were processed in OriginLab software.

VIS-emission spectra were recorded with Jasco FP-8300 spectrofluorometer in 10 mm path cuvette (Hellma Analytics 115-F-10-40).

Raman measurements were recorded on a Renishaw inVia™ microscrope at room temperature. SWCNT samples were drop-casted on a glassbottom petridish (ibidi µ-Dish 35mm #1.5 (170 µm +/- 5µm) glass bottom). Raman spectra were obtained by exciting SWCNT films with a 532nm laser (Renishaw RL-08) set to 100% power and mapping an area of 80 x 80 points with a 5x objective.

The recoded spectra were averaged and baseline corrected using the manufacturers software. Further analysis was performed in Python 3.12 using numpy, scipy and matplotlib. The observed D and G modes were fitting with Lorentzian functions, and the D/G ratio was calculated from the respective integrated areas.

2. Synthesis of 7-diazo-4-methylcoumarin tetrafluoroborate (CoumNN)

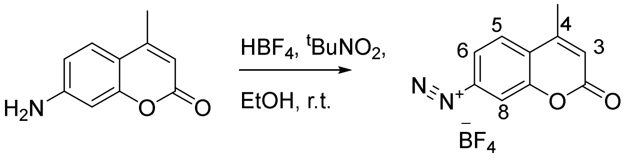

In a 25 ml glass vial equipped with stirring bar 7-amino-4-methylcoumarin (**$\mathbf{CoumNH_2}$**,17.5 mg, 100 µmol) was suspended in 500 µL of ethanol. Then, under stirring at 0 °C, tetrafluoroboric acid (20 µL, 50%aq., 1.1 eq, 114 µmol) was added to the solution dropwise within 1 min. Afterwards, *tert*-Butyl nitrite (20 µL, 1.7 eq, 168 µmol) was added dropwise at 0 °C within 5 min. The reaction mixture was stirred at r.t. for 30 min. Then, 4 ml of diethyl ether was added to the reaction, the suspension was transferred into 4 2 mL Eppendorf tube and centrifuged at 16100 *g* for 5 min. Afterwards, the supernatant was discarded, and the

new 4 mL portion of diethyl ether was added. The process was repeated three times till the supernatant solution was colorless after centrifugation. The residue was dried under vacuum resulting in 14 mg of colorless powder (78%).

^{1}H-NMR (300 MHz, CD3CN) δ 8.41 – 8.29 (m, 2H, $H^{6,8}$), 8.16 (dd, *J* = 8.7, 2.9 Hz, 1H, H^5), 6.70 (d, *J* = 1.7 Hz, 1H, H^3), 2.52 – 2.49 (d, *J* = 1.4 Hz, 3H, C$\underline{H_3}$).

^{13}C-NMR (75 MHz, CD_3CN) δ 151.94, 132.16, 129.92, 127.99, 122.51, 122.37, 120.88, 18.50.

3. Synthesis of arylazosulfonates

*3.1. Synthesis of 4-methylcoumarin sodium 7-diazenesulfonate (**CoumNNS**)*

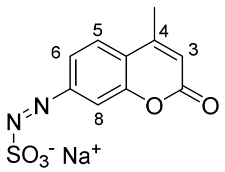

Na_2SO_3 (3.15 mg, 25 µmol or 6.3 mg, 50 µmol) was dissolved in 5 mL of MQ water resulting in 5 mM or 10 mM stock solutions. In 2 ml Eppendorf tube **CoumNN** (0.9 mg, 5 µM) was dissolved in freshly prepared Na_2SO_3 solution (10 mM or 5 mM). The resulting yellow solutions of 5 mM of **CoumNNS** or **CoumNNS***, respectively were immediately used for the QD introduction reactions. **Sample for NMR** studies was prepared prior to the measurement as follows. **CoumNN** (5 mM) was dissolved in D_2O containing Na_2SO_3 (10 mM) resulting in 5 mM of **CoumNNS**. NMR spectra were recorded on a Brucker AV-300 spectrometer at 298 K within an hour after the sample preparation. Recorded spectra were analyzed with the MestReNova software (Figures S2-S5). ^{13}C-NMR *J*-modulated spin-echo (JMOD) spectrum revealed five (negative) resonances, which correlate with the quaternary carbons of **CoumNNS** (δ_C 163.05, 154.72, 152.49, 150.70, 123.45). The negative values are to be expected for JMOD spectra of quaternary carbons and CH_2- groups.

^{1}H-NMR (300 MHz, D_2O) δ 7.98 (d, *J* = 8.4 Hz, 1H, H^8), 7.92 – 7.79 (m, 2H, $H^{5,6}$), 6.52 (d, *J* = 1.3 Hz, 1H, H^3), 2.53 (d, *J* = 1.3 Hz, 3H, C$\underline{H_3}$).

^{13}C-NMR JMOD (75 MHz, D_2O) δ 163.05, 154.72, 152.49, 150.70, 126.62, 123.45, 119.65, 115.65, 110.89, 18.03.

*3.2. Synthesis of p-nitroaryl sodium diazenesulfonate (**NO_2ArNNS**)*

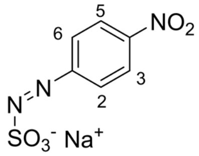

Na_2SO_3 (6.3 mg, 50 µmol) was dissolved in 5 mL of MQ water resulting in 10 mM stock solution. In 2 ml Eppendorf tube *P*-diazo-nitroaryl tetrafluoroborate (**NO_2ArDz**, 1.18 mg, 5 µmol) was dissolved in 1 mL of freshly prepared Na_2SO_3 solution (10 mM). The resulting yellow solutions of 5 mM of **NO_2ArNNS** was immediately used for the QD introduction reactions. **Sample for NMR** studies was prepared prior to the measurement as follows. **NO_2ArDz** (5 µmol) was dissolved in D_2O containing Na_2SO_3 (10 mM) resulting in 5 mM of **NO_2ArNNS**. NMR spectra were recorded on a Brucker AV-300 spectrometer at 298 K within an hour after the sample preparation. Recorded spectra were analyzed with the MestReNova and TopSpin softwares (Figures S7-S10). ^{13}C and ^{1}H-^{13}C HSQC spectra revealed the chemically equivalent protons 3/5 and 2/6, which possess the same ^{1}H and ^{13}C NMR frequencies. Peaks 154.71 and 139.00 of the ^{13}C-NMR correlate to the quaternary carbons C^1 and C^4, respectively.

^{1}H-NMR (300 MHz, D_2O) δ 8.18 (dt, *J* = 3.07, 9.40, 1.91, 2H, $H^{2,6}$) 7.08 (dt, *J* = 3.07, 9.40, 1.91, 2H, $H^{3,5}$).

^{13}C-NMR (75 MHz, D_2O) δ 154.71, 139.00, 126.36, 112.04

4. (6,5) - SWCNT sample preparation

CoMoCAT (6,5)-chirality enriched SWCNTs dispersed in MQ water (2 mg/ml) were mixed with sodium dodecylbenzenesulfonate (SDBS, 2% w/v) in 1:1 (v/v) ratio. The resulting mixture was transferred to Eppendorf tubes and sonicated in a cup-horn sonicator (Fisher Scientific Model 120 Sonic Dismembrator, 55% amplitude, 40 min, 10 ℃). The obtained suspension was then centrifuged (2x16000 *g*, 30 min) and separated from the formed pellet, resulting in a colloidally stable dispersion. The resulting dispersion was kept in the dark at 4 ℃ and used for further sp^3 defect (quantum defects, **QDs**) introduction reactions.

The concentration of the prepared sample was estimated from NIR absorption spectra according to the previously established procedure by the formula: [2]

$$c_C = B\frac{\Delta_{fwhm}OD}{fd},$$

c_C is carbon atom concentration in suspension [mol/L], Δ_{fwhm} is the line width at half maximum [nm], *OD* is the optical density at 1000 nm, B = 5.1 x 10^{-8} $mol.L^{-1}.cm.nm^{-1}$, *d* is the thickness of the optical cell, *f* is the atom oscillator strength = 0.01.

To convert the carbon atom concentration into the SWCNT's concentration we used the formula:

$$SWCNT_C = \frac{c_C}{c_{SWCNT} \times 10^9},$$

$SWCNT_C$ is the (6,5) – SWCNT concentration and $c_{SWCNT} = SWCNT\ length \times c\ per\ nm$ is the carbon concentration per nanotube, $SWCNT\ length = 600\ nm$, $c\ per\ nm$ = 88.

5. sp^3 quantum defect introduction with diazonium salts

Introduction of sp^3 quantum defects (**QDs**) via a photoreaction with diazonium salts was performed using a previously described method.[3,4] In short, freshly prepared diazonium salt solution was added to the (6,5)-SWCNT suspension (10 nM, 1.6 OD at 1000 nm), followed by 527 nm LED green light irradiation during various times controlled by NIR emission and absorption spectroscopy.

*5.1. Coumarin-based sp^3 QD introduction (photoreaction with **CoumNN**)*

7-diazo-4-methylcoumarin tetrafluoroborate (**CoumNN**, 1 mg, 5 µmol) was dissolved in 1 mL of MQ water to obtain a 5 mM stock solution. Previously prepared (6,5)-SWCNT suspension in MQ water (10 nM, 1.6 OD at 1000 nm, 1% SDBS) was distributed into 96 well-plate. Then, the specified amount of **CoumNN** stock (2, 4 or 8 µL) were added to the wells resulting in 10 or 50 µM of final concentration in the well, respectively. The 96-well plate was irradiated with a 527 nm LED at 25 mA for 15, 30, 45, 60, 75 and 90 min. NIR emission spectra were taken after each period of irradiation.

CoumNN, (1 mg, 5 µmol) was dissolved in 1 mL of MQ water to obtain a 5 mM stock solution. Previously prepared (6,5)-SWCNT suspension in MQ water (10 nM, 1.6 OD at 1000 nm, 1% SDBS) was distributed into 96 well-plate. Then, the specified amount of **CoumNN** stock (2, 4 or 8 µL) were added to the wells resulting in 20, 30 or 40 µM of final concentration in the well, respectively. The 96-well plate was irradiated with a 527 nm LED at 25 mA for 30 min. The samples were scanned with the NIR emission spectrometer irradiated with 561 nm laser at 100 mW. Recorded data were processed with custom Python script.

*5.2. Nitro-aryl-based sp^3 QD introduction (photoreaction with **NO_2ArDz**)*

P-diazo-nitroaryl tetrafluoroborate (**NO_2ArDz**, 1.18 mg, 5 µmol) was dissolved in 1 mL of MQ water resulting in a 5 mM stock solution. Previously prepared (6,5)-SWCNT suspension in MQ water (10 nM, 1.6 OD at 1000 nm, 1% SDBS) was distributed into 96 well-plate. Then, 2 uL of **NO_2ArDz** stock was added to the wells resulting in 10 µM of final concentration in the well. The 96-well plate was the irradiated with 527 nm LED at 25 mA during 15, 30, 45, 60, 75 and 90 min. NIR emission spectra were taken after each period of irradiation.

6. Na_2SO_3 - assisted sp^3 quantum defect introduction

*6.1. sp^3 QD introduction under green light with **CoumNNS** prepared in situ*

The previously prepared (6,5) – enriched SWCNT dispersion in 1% SDBS MQ water was diluted with 1% SDBS solution containing Na_2SO_3 resulting in 10 nM (6,5) – SWCNT - 1% SDBS solution with 1-5 mM of Na_2SO_3 was distributed into 96 well-plate. Then, the specified amounts of freshly prepared **CoumNN** stock (2, 4 or 8 µL) were added to the wells resulting in 50, 100 or 200 µM of *in situ* – formed **CoumNNS** final concentration in the well, respectively. The 96-well plate was then irradiated with the 527 nm LED at 25 mA during 15, 30, 45, 60, 75 and 90 min. NIR emission spectra were taken after each period of irradiation.

*6.2. sp^3 QD introduction under 375 nm and 470 nm LED with **CoumNNS** prepared prior to the photoreaction*

(6,5)-SWCNTs were dispersed in SDBS (1%, w/w) (1.5 mL) and then placed in a petri dish. **CoumNN** (0.94 mg) was dissolved in 10 mM MQ water solution of Na_2SO_3 (6.3 mg in 5 mL, 2 eq) forming the yellow solution of 5 mM of **CoumNNS**. To the solution in the petri dish the solution of **CoumNNS** (3 µL; final concentration = 10, 40 or 250 µM) was then added at once and mixed well. The reaction solution was placed under the respective LED light and irradiated for 1.5 h. Samples of 10 µL were taken every 15 min and placed into the 96 well-plate. In between the measurements the well-plate was screened from light. The distributed this way samples in the well-plate were then diluted till 200 µL per well with SDBS solution (1%, w/w) and scanned with the NIR emission spectrometer irradiated with 561 nm laser at 100 mW. Recorded data were processed with a custom Python script.

*6.3. sp^3 QD introduction under green light with **CoumNNS** prepared prior to the photoreaction*

Previously prepared (6,5) – enriched SWCNT dispersion in 1% SDBS MQ water was diluted with 1% SDBS solution resulting in 10 nM (6,5) – SWCNT - 1% SDBS solution. **CoumNN** (0.94 mg) was dissolved in 10 mM MQ water solution of Na_2SO_3 (6.3 mg in 5 mL, 2 eq) forming a yellow solution of 5 mM of **CoumNNS**. Then **CoumNNS** (8 µL; final concentration = 40 µM) was added to the (6,5) – SWCNTs solution. 100 µL of the reaction mixture was added to the well and irradiated with 25 mA 561 nm LED light for 15 min. The next well was prepared identically and the whole plate was irradiated for 15 minutes more, resulting in 2 samples irradiated for 15 and 30 min. The procedure was repeated until there were 8 wells irradiated for 15, 30, 45, 60, 75 and 90 min, respectively. The wells were filled up with 100 µL of the new 1%-SDBS solution (200 µL per well in total) and measured with the NIR emission spectrometer irradiated with 561 nm laser at 100 mW. Recorded data were processed with custom Python script.

*6.4. sp^3 QD introduction under green light with **NO_2ArNNS** prepared in situ*

Previously prepared (6,5) – enriched SWCNT dispersion in 1% SDBS MQ water was diluted with 1% SDBS solution containing Na_2SO_3 resulting in 10 nM (6,5) – SWCNT - 1% SDBS solution with 1-5 mM of Na_2SO_3 was distributed into 96 well-plate. Then, the specified amounts of freshly prepared **NO_2ArDz** 5 mM stock (2, 4 or 8 µL) were added to the wells resulting in 50, 100 or 200 µM of in situ – formed **NO_2ArNNS** final concentration in the well, respectively. The 96-well plate was then irradiated with the 527 nm LED at 25 mA during 15, 30, 45 and 60 min. NIR emission spectra were taken after each period of irradiation. Recorded data were processed with custom Python script.

7. Mechanistic experiments

7.1. Photoreaction in the presence of TEMPO under green light

15 min after introduction of the coumarin-based sp^3 QD (see 5.2 section) 2,2,6,6-tetramethylpiperidine-1-oxy radical (**TEMPO**, 2 eq) was added to the well. The 96-well plate was then irradiated with 527 nm LED at 25 mA for 15, 30, 45, 60, 75 and 90 min. NIR emission spectra were taken after each period of irradiation. Experiments were performed as triplicates.

7.2. Photoreaction in the presence of TEMPO under 375 nm UV light

In 15 min after introduction of the coumarin-based sp^3 QD (see 5.2 section) 2,2,6,6-tetramethylpiperidine-1-oxy radical (**TEMPO**, 3 eq) was added to the petri dish. The sample was continuously irradiated with 375 nm LED light. Samples were taken every 15 min and placed onto the 96 well-plate. In between the measurements the well-plate was screened from light. The distributed this way samples in the well-plate were then diluted till 200 µL per well with SDBS solution (1%, w/w) and scanned with the NIR emission spectrometer irradiated with 561 nm laser at 100 mW. Recorded data were processed with custom Python script.

7.3. Control reaction with Na_2SO_3 and an amino-derivative of coumarin under green light irradiation

To exclude the possibility of forming oxygen defects with Coumarine, we have performed the reaction with amino precursor. **CoumNH$_2$** (see 2 section) in the same conditions as the established introduction of the sp^3 quantum defects via radical reaction with diazonium salts (see 5.1 section). For that the previously prepared (6,5) – enriched SWCNT dispersion in 1% SDBS MQ water was diluted with 1% SDBS solution containing Na_2SO_3 resulting in 10 nM (6,5) – SWCNT - 1% SDBS solution with 5 mM of Na_2SO_3 was distributed into 96 well-plate. Then, the specified amounts of freshly prepared **CoumNH$_2$** 5 mM stock solution (1.6, 2, 4, 6 or 8 µL) were added to the wells resulting in 40, 50, 100, 150 or 200 µM of **CoumNH$_2$** final concentration in the well, respectively. The 96-well plate was then irradiated with the 527 nm LED at 25 mA during 30 min). The well-plate was scanned with the NIR emission spectrometer irradiated with 561 nm laser at 100 mW. Recorded data were processed with custom Python script.

7.4. Sample preparation for NMR in deuterated water with and without Na_2SO_3

To investigate the coumarin derivatives formed in aqueous conditions subsequently to the sp^3 QDs introduction into the (6,5) – SWCNTs' surface, we imitated the reactions conditions prior to the addition of the nanotubes in the NMR tube and analyzed the resulted ^{1}H-NMR spectra. For that we dissolved **CoumNN** (5 mM) in D_2O and in D_2O containing Na_2SO_3 (10 mM) resulting in 2 NMR samples of 5 mM of **CoumOH** and 5 mM of **CoumNNS** (Figure S6). ^{1}H-NMR spectra were recorded on a Brucker AV-300 spectrometer at 298 K. Recorded spectra were analyzed with the MestReNova software.

CoumOH

^{1}H NMR (300 MHz, D_2O) δ 7.66 (d, *J* = 8.8 Hz, 1H), 6.92 (dd, *J* = 8.8, 2.4 Hz, 1H), 6.82 (d, *J* = 2.4 Hz, 1H), 6.19 (d, *J* = 1.1 Hz, 1H), 2.43 (d, *J* = 1.1 Hz, 3H). The spectrum corresponds to literature data [5,6]

CoumNNS see section 3.1.

8. MeOH and ^{i}PrOH - assisted surfactant exchange to ss-DNA and PEG-PL

The procedure is based on the protocol described previously. [7] In short, 200 µL of 1 % SDBS (6,5)-CCNT solution was mixed with 20 µL of aqueous buffered ss-DNA solution ($(GT)_{10}$ or $(GT)_{15}$, 100 µM, PBS, pH = 7.4) or PEG-PL solution (2 mg/mL, PBS, pH = 7.4) and 10 µL of 40% (m/v) PEG6K solution. Then 180 µL of methanol was added dropwise while the tube was vortexed. Afterwards, 530 µL of isopropyl alcohol was added in 2 portions. After appearance of SWCNT aggregates the suspension was centrifuged for 2 min at 16100g. The supernatant was discarded, solvents remnants were evaporated on air, and the obtained pellet was redispersed in PBS (200 µL, pH = 7.4). The obtained solution underwent the protocol for the second time and after the second redispersion was tip-sonicated at 25% amplitude for 20 s. The final suspension was then centrifuged for 5 min. The obtained supernatant was collected and used for the evaluation.

9. Dopamine, epinephrine and riboflavin response studies

In 96 well plate previously $(GT)_{15}$-(6,5)-CCNTs were distributed to 9 wells (approx. 0.2 nM, PBS, pH = 7.4, 200 µL). The samples were measured with the NIR emission custom-built setup described above. These spectra were later used for calculation of the I_0 overall emission. Freshly prepared solutions of dopamine, epinephrine and riboflavin (all 5 mM, PBS, pH = 7.4) were added to the wells each in triplicate (each 2 µL,

100 µM of final concentration in a well). The samples were measured as the same settings as before the addition of the analytes. Recorded data were processed with custom Python script.

Supplementary figures

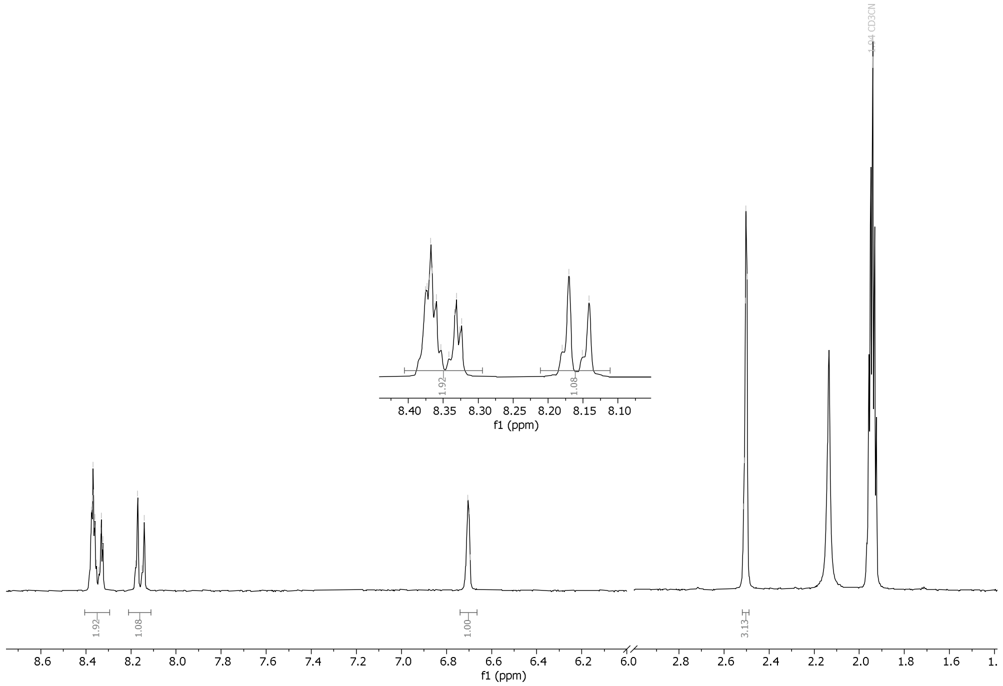

Figure S1. ^{1}H-NMR spectrum of 7-diazo-4-methylcoumarin tetrafluoroborate in acetonitrile-d_3.

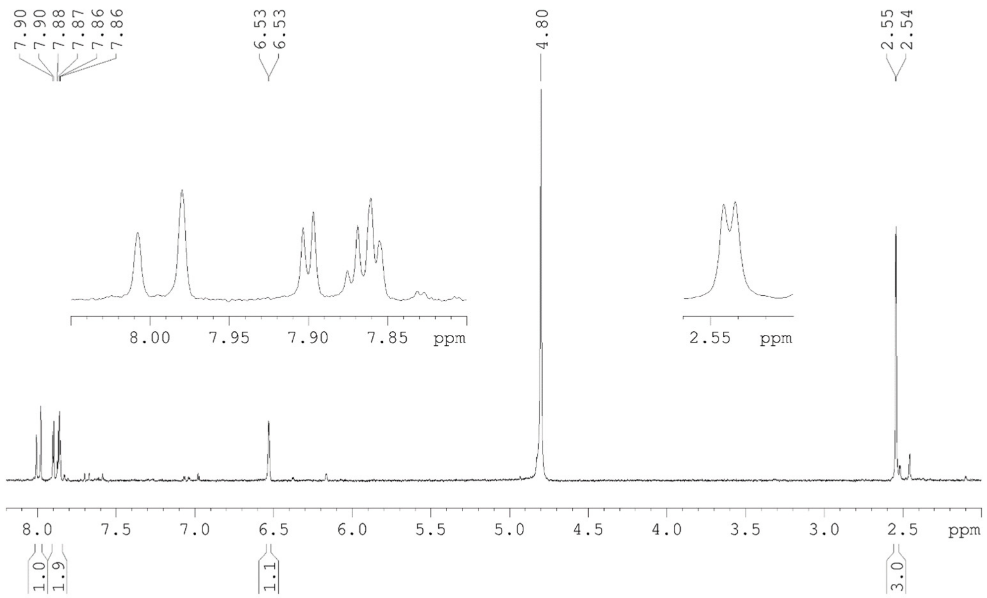

Figure S2. ^{1}H-NMR spectrum of 4-methylcoumarin sodium 7-diazenesulfonate (**CoumNNS**) in D_2O.

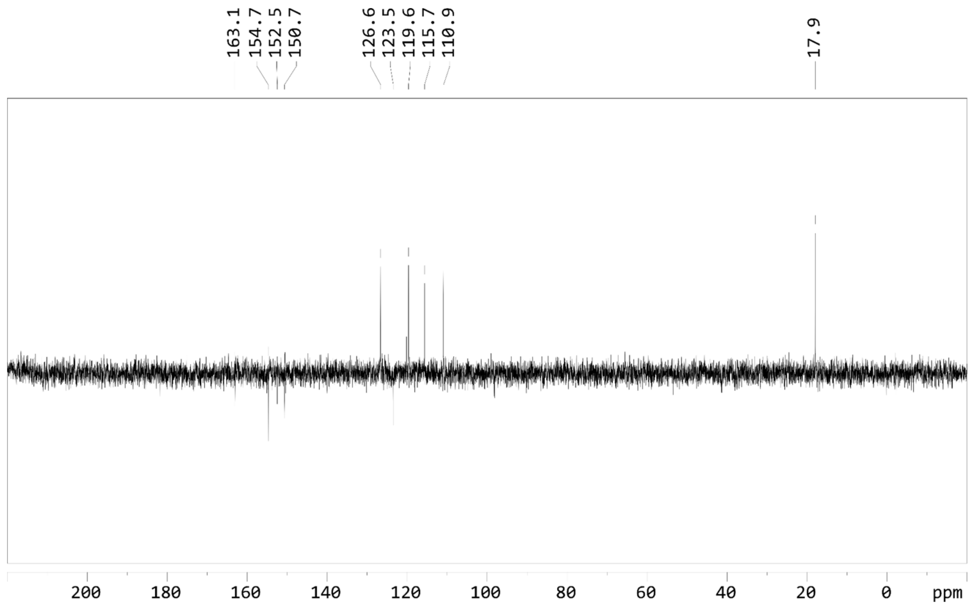

Figure S3. ^{13}C-NMR *J*-modulated spin-echo (JMOD) spectrum of 4-methylcoumarin sodium 7-diazenesulfonate (**CoumNNS**) in D_2O.

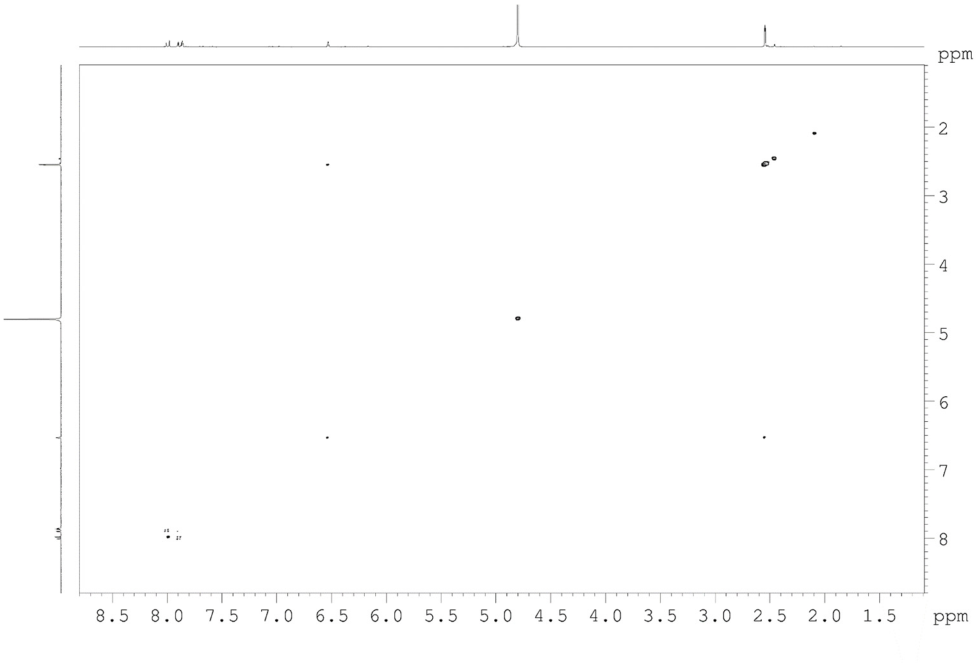

Figure S4. ^{1}H-COSY-NMR spectrum of 4-methylcoumarin sodium 7-diazenesulfonate (**CoumNNS**) in D_2O.

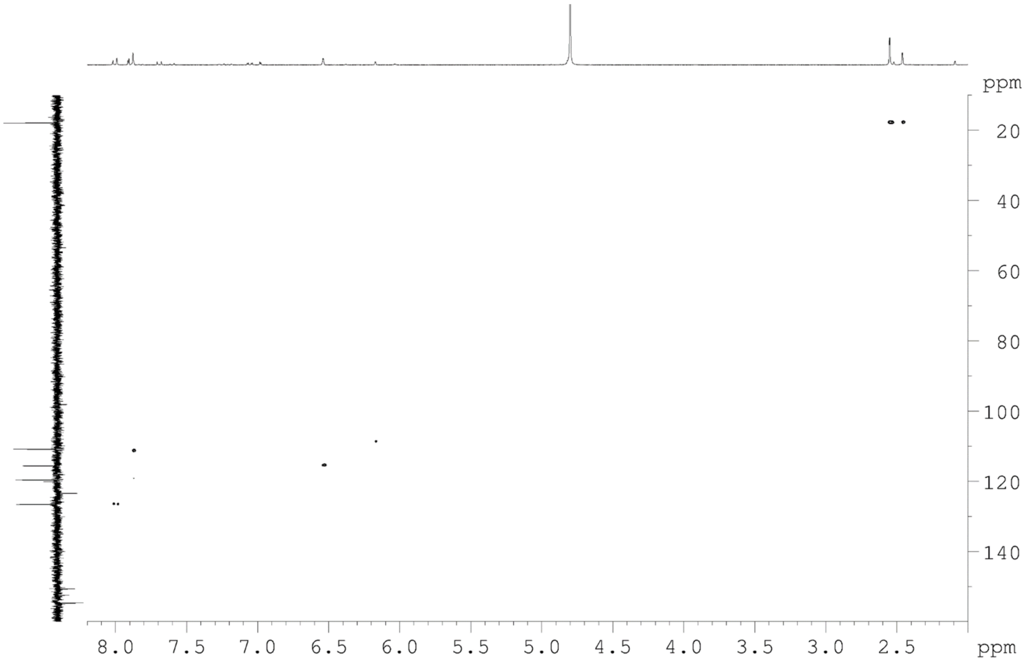

Figure S5. ^{1}H-^{13}C-HSQC-NMR spectrum of 4-methylcoumarin sodium 7-diazenesulfonate (**CoumNNS**) in D_2O.

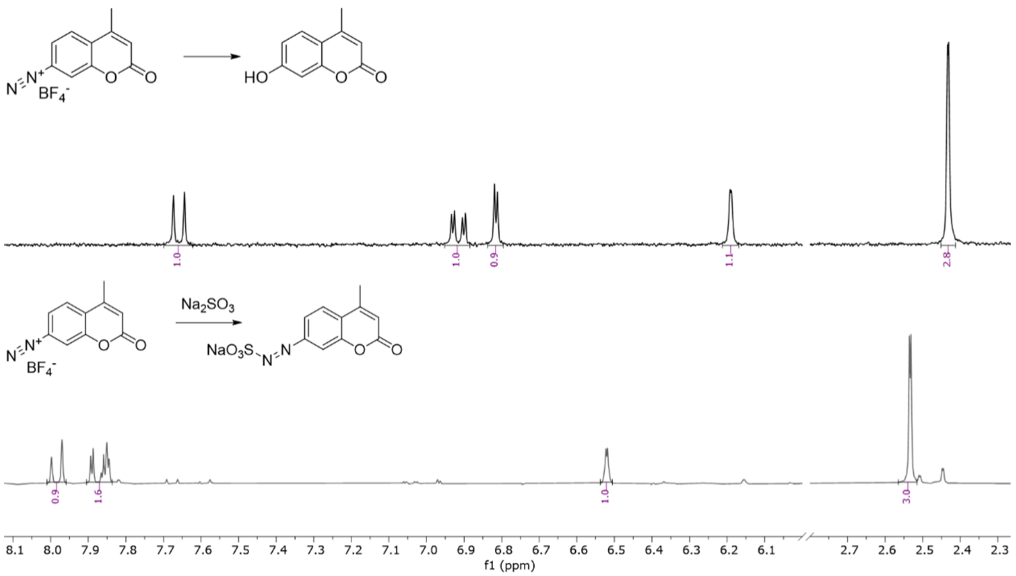

Figure S6. Comparison of the ^{1}H-NMR spectra of 7-hydroxy-4-methylcoumarin (top) and 4-methylcoumarin-7-diazene-1-sulfonate sodium salt in D_2O (bottom).

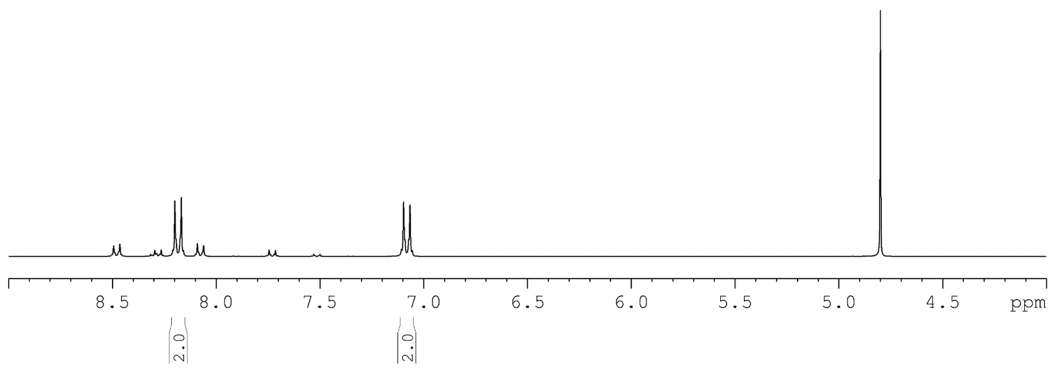

Figure S7. ^{1}H-NMR spectrum of 4 p-nitroaryl sodium diazenesulfonate (**NO_2ArNNS**) in D_2O.

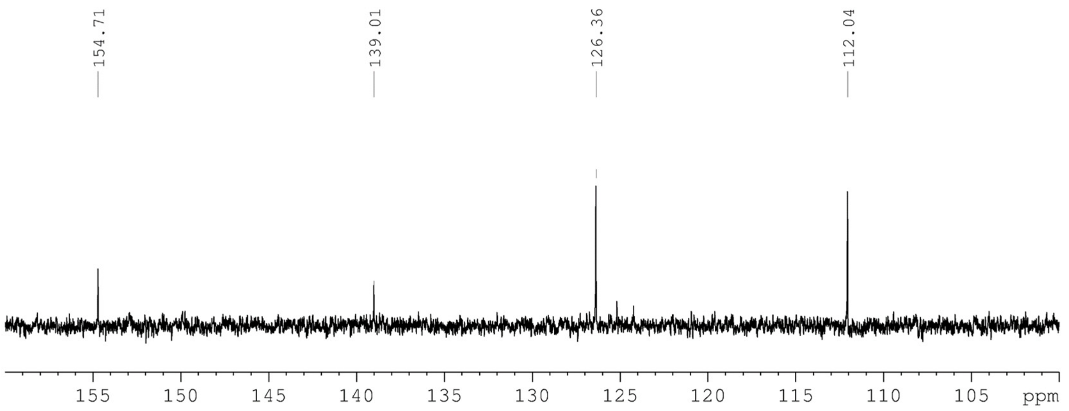

Figure S8. ^{13}C-NMR spectrum of 4 p-nitroaryl sodium diazenesulfonate (**NO_2ArNNS**) in D_2O.

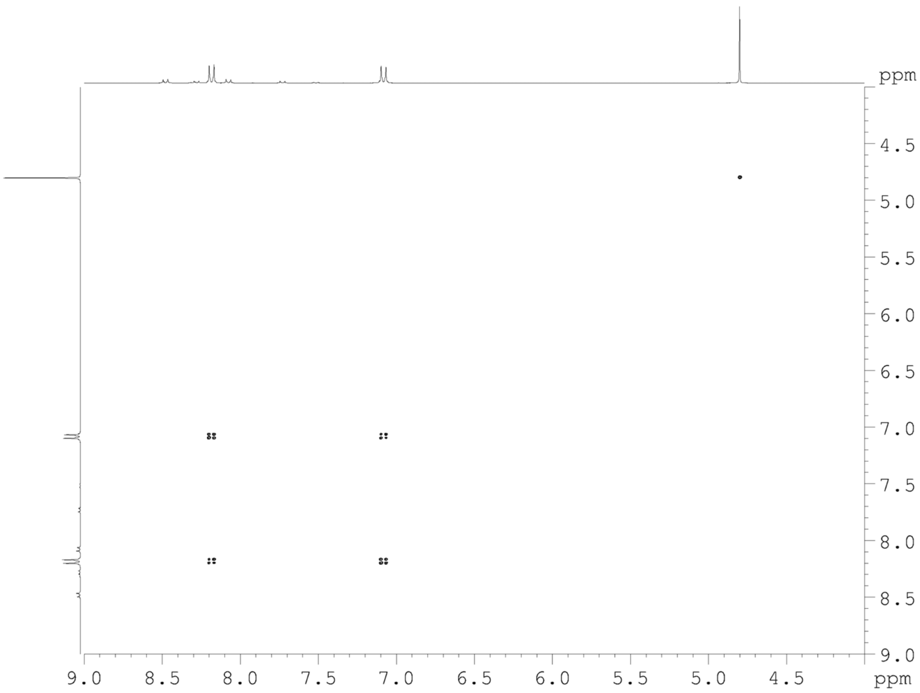

Figure S9. ^{1}H-COSY-NMR spectrum of 4 p-nitroaryl sodium diazenesulfonate (**NO_2ArNNS**) in D_2O.

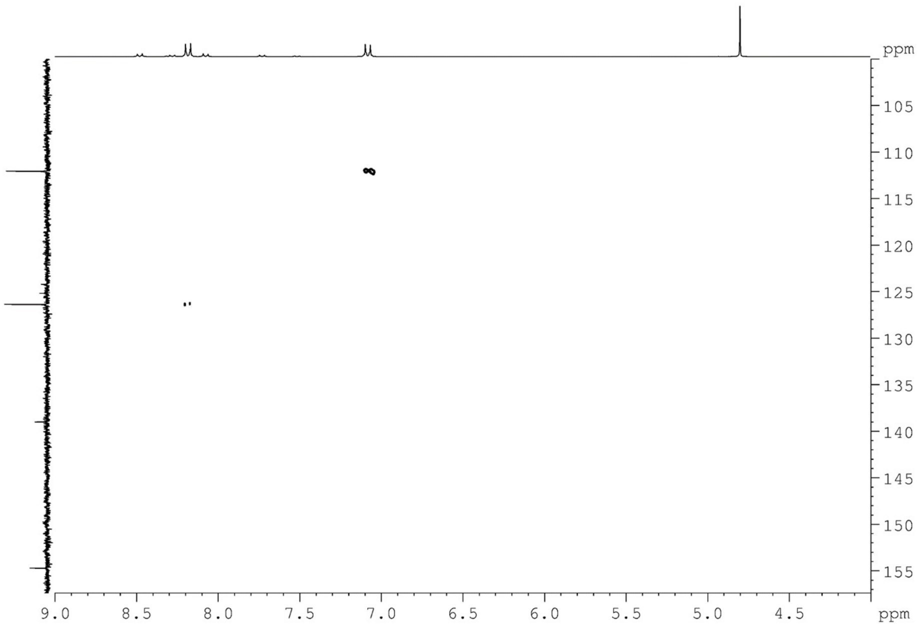

Figure S10. ^{1}H-^{13}C-HSQC-NMR spectrum of 4 p-nitroaryl sodium diazenesulfonate (**NO_2ArNNS**) in D_2O.

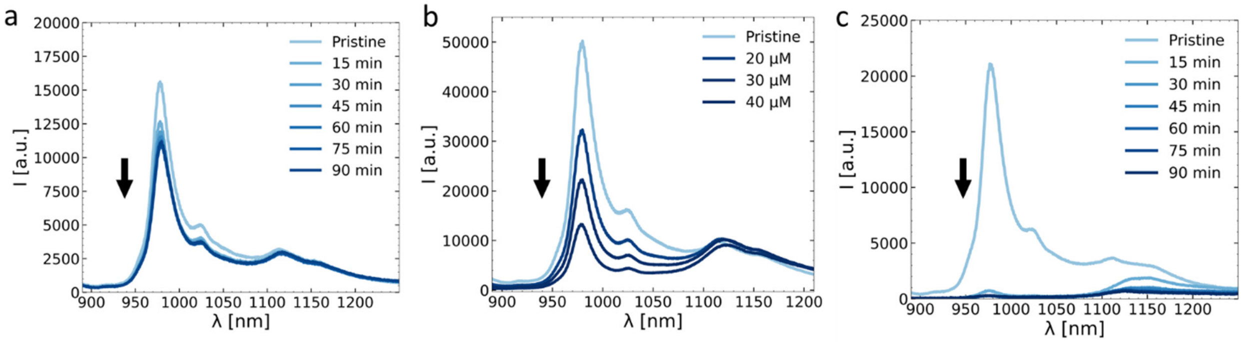

Figure S11. E_{11} quenching in the presence of different CoumNN concentrations. a) 5 µM; b)20-40 µM; c) 50 µM.

Non-normalized emission spectra of SDBS-(6,5)-SWCNTs after introduction of sp^3 QDs by the conventional method with **CoumNN** at different concentrations of the diazonium salt (0 µM = pristine, 20 µM, 30 µM, 40 µM). The samples were irradiated with 561 nm LED light at 25 mA. In this experiment freshly-prepared **CoumNN** solution in MQ water was added to the 10 nM solution of the (6,5)-SWCNTs suspended in 1% SDBS surfactant in distilled water, followed by green light irradiation (527 nm).

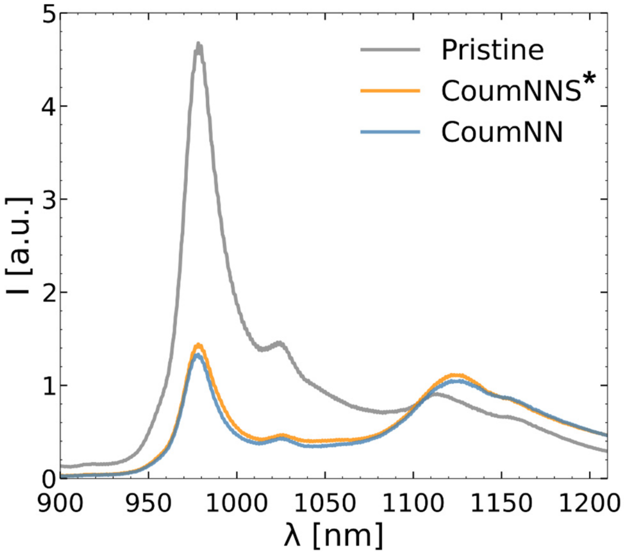

Figure S12. NIR emission of SDBS-(6,5)-CCNTs samples prepared for Raman by coumarin-based QD introduction via the photoreaction with 40 µM of **CoumNN** under 527 nm LED (yellow) and with 50 uM of **CoumNNS*** prepared under the 375 nm LED (blue) irradiation. **CoumNNS*** is in advance prepared **CoumNNS** in the deficit of Na_2SO_3 (mixed in 1:1 molar ratio) and thus still containing **CoumNN**.

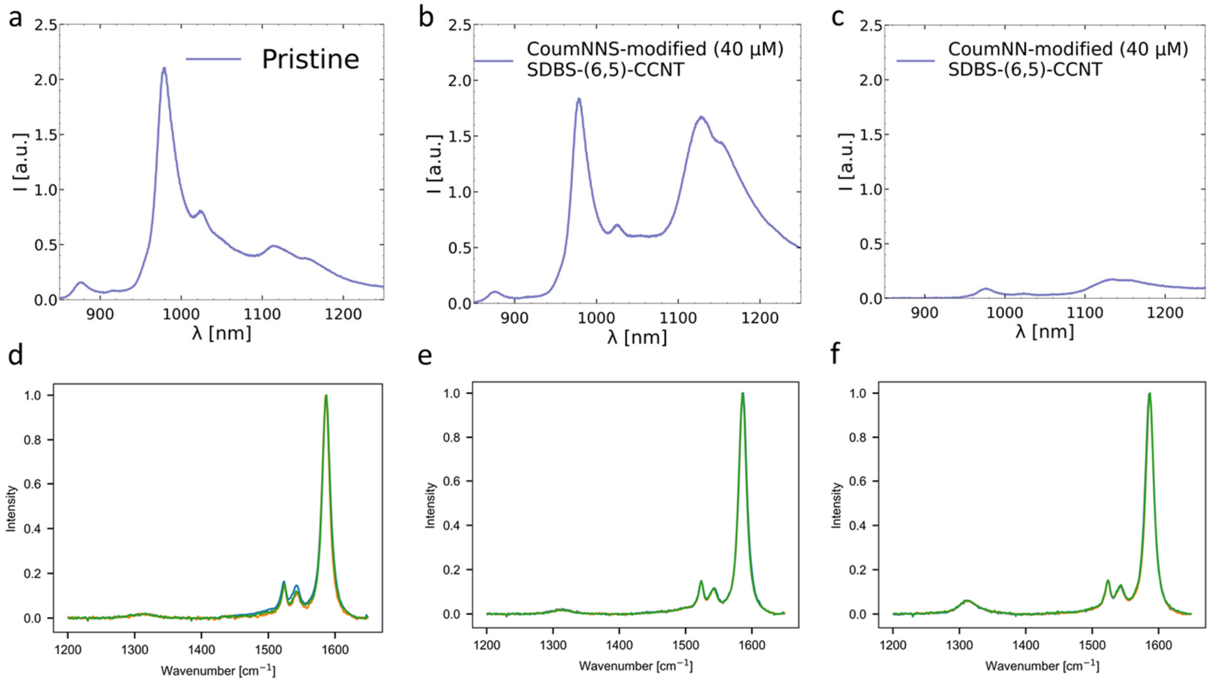

Figure S13. Emission and Raman spectra of a,d) Pristine SDBS-(6,5)-SWCNTs *(D/G = 0.045 ± 0.006, mean ± SE, n=3)*, b,e) **CoumNNS** (40 µM, see section 6.2.)- modified SDBS-(6,5)-CCNTs *(D/G = 0.042 ± 0.004, mean ± SE, n=3)*, and c,f) **CoumNN** (40 µM, see section 5.1.)- modified SDBS-(6,5)-CCNTs *(D/G = 0.125 ± 0.004, mean ± SE, n=3)*. All the emission spectra were made at the same settings and the same SWCNT concentration of 10 nM. Orange, blue and green spectra in d, e and f correspond to 3 replicates.

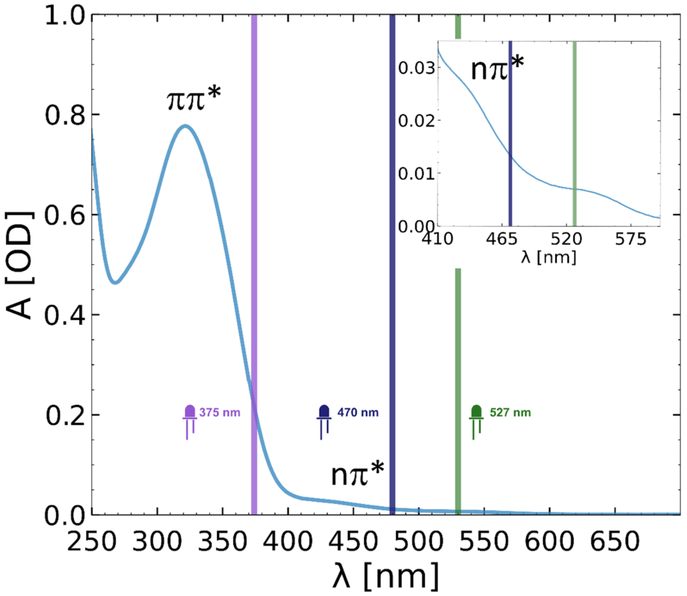

Figure S14. Absorption spectrum of freshly-prepared **CoumNNS** in water.

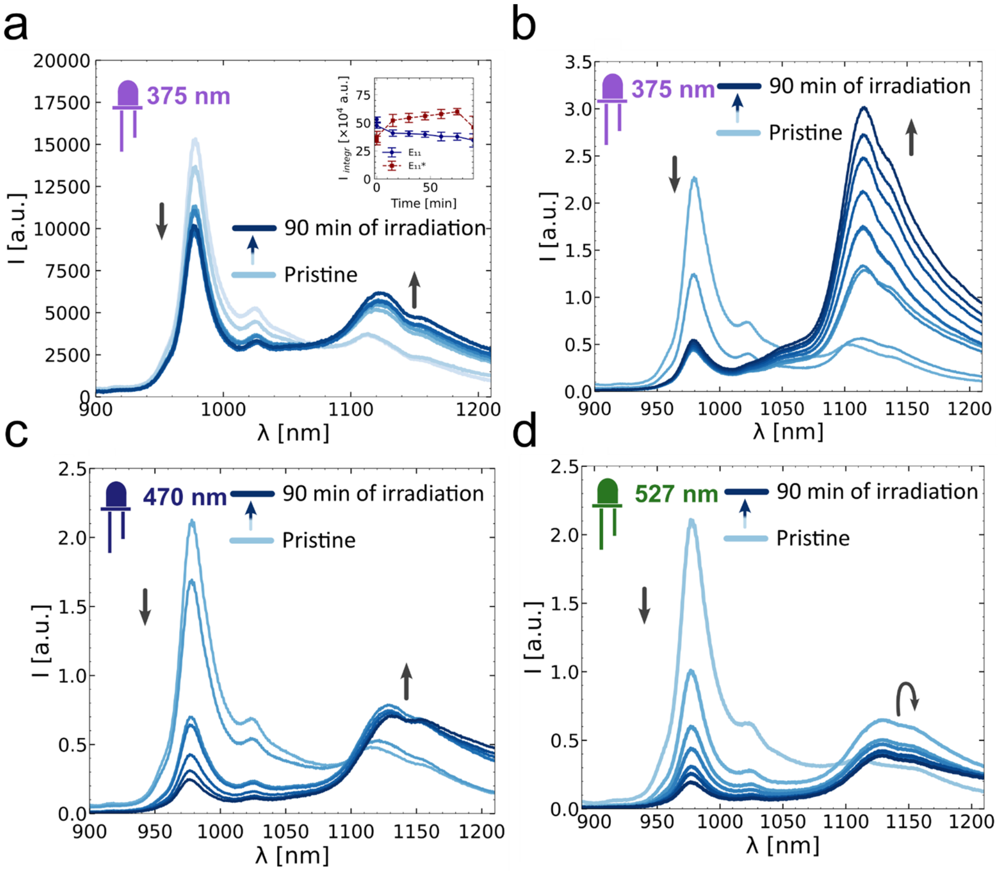

Figure S15. Photoreaction of SDBS-(6,5)-SWCNTs in in MQ water with **CoumNNS** and **CoumNNS*** prepared prior to the addition to the SWCNT sample. a) Photoreaction with 10 µM of **CoumNNS** under 375 nm UV LED light (Inset: mean values (n=3) of E_{11} (blue) and E_{11}* (red) ±SE). b) Photoreaction with 250 µM of **CoumNNS*** under 375 nm LED light. c) Photoreaction with 250 µM of **CoumNNS*** under 470 nm blue light. d) Photoreaction with 200 µM of **CoumNNS*** under 470 nm blue light.

CoumNNS* is in advance prepared **CoumNNS** in the deficit of Na_2SO_3 (mixed in 1:1 molar ratio) and thus still containing **CoumNN**. E_{11}* in (d) is first increased above the emission at the same wavelength in pristine nanotubes sample and then decreases overtime most probably due to physisorption of the **CoumOH** species formed from the unreacted with Na_2SO_3 part of **CoumNN**.

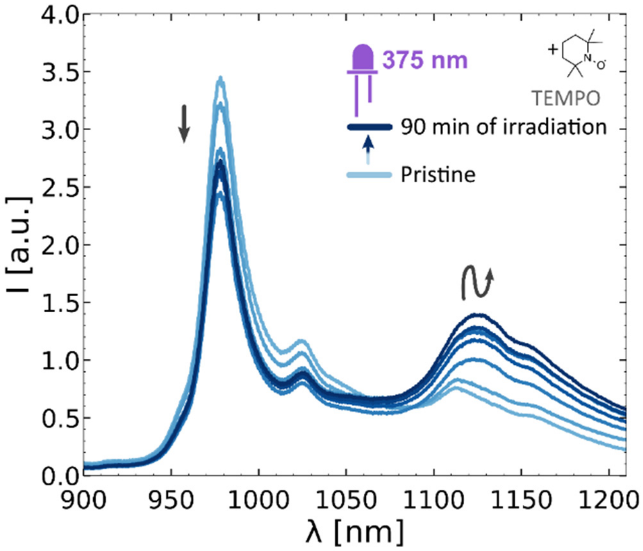

Figure S16. Photoreaction of (6,5)-SWCNTs dispersed in 1% SDBS (10 nM) with **CoumNNS** in the presence of **TEMPO** under 375 nm LED light.

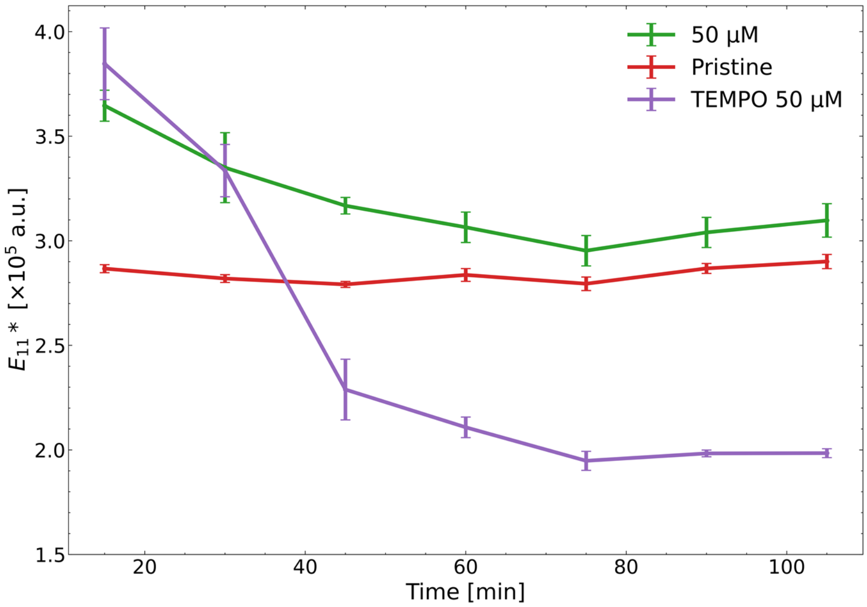

Figure S17. Photoreaction of (6,5) – SWCNTs dispersed in 1% SDBS in MQ solution with **CoumNNS** prepared prior to the addition to the nanotubes (1:1 ratio of the Na_2SO_3 to the **CoumNN**) sample under 527 nm UV LED light (green), with added 2 equivalents of TEMPO after 15 min from the beginning of reaction (purple), and the control pristine (6,5) – SDBS – SWCNTs without any additional reagents (red). All the values are median ± SE, n = 3.

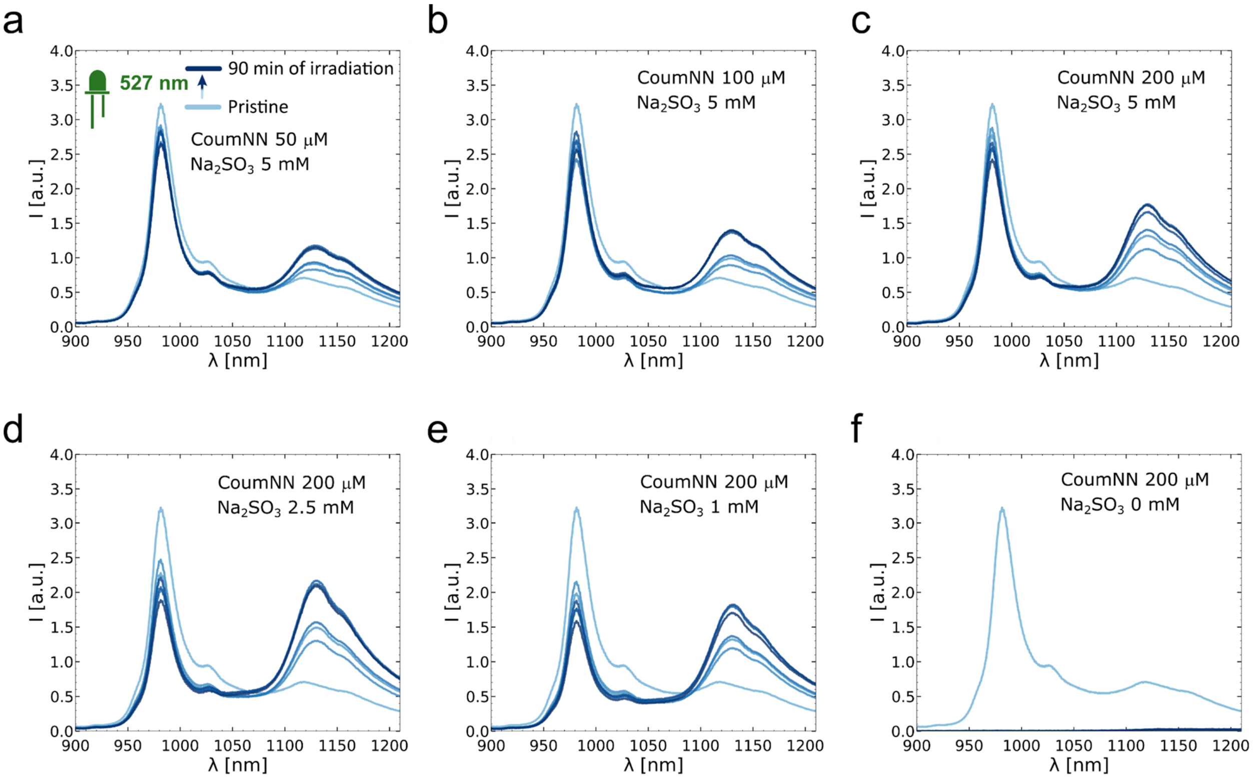

Figure S18. Emission spectra of samples of different one pot photoreactions of SDBS-(6,5)-SWCNTs with **CoumNNS. CoumNNS** formed *in situ* in the solution of (6,5)–SDBS-SWCNTs containing Na_2SO_3 at various concentrations of **CoumNN** and Na_2SO_3 combinations irradiated with 527 nm green LED light. a) **CoumNN** added = 50 µM, Na_2SO_3 = 5 mM. b) **CoumNN** added = 100 µM, Na_2SO_3 = 5 mM. c) **CoumNN** added = 200 µM, Na_2SO_3 = 5 mM. d) **CoumNN** added = 200 µM, Na_2SO_3 = 2.5 mM. e) **CoumNN** added = 200 µM, Na_2SO_3 = 1 mM. f) Control measurement in the absence of Na_2SO_3 (**CoumNN** added = 200 µM, Na_2SO_3 = 0 mM).

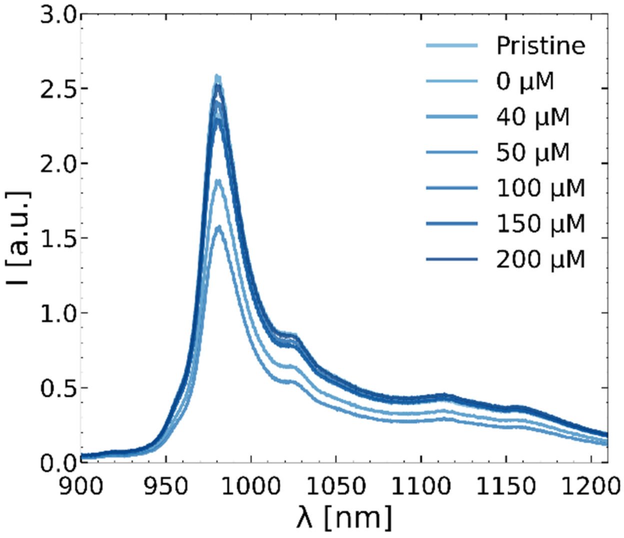

Figure S19. Control reaction of (6,5)-SWCNTs dispersed in 1% SDBS in MQ with 5 mM of Na_2SO_3 and an amino-derivative of coumarin (**CoumNH₂**) under green LED light irradiation.

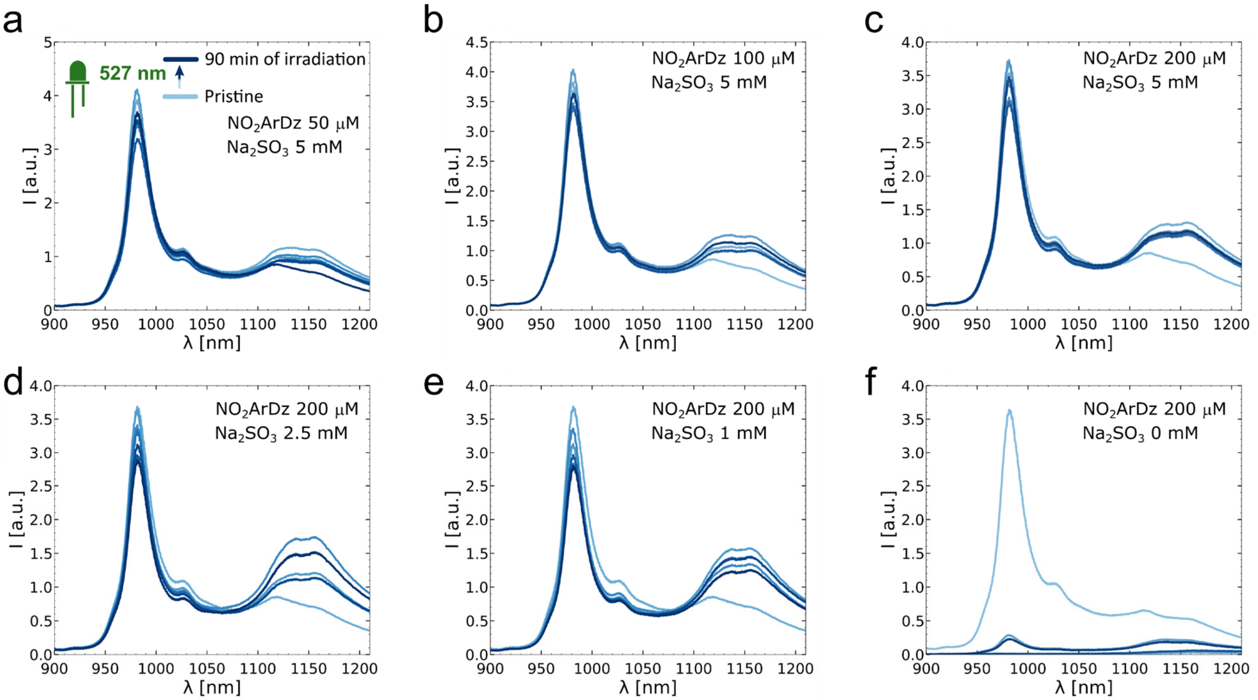

Figure S20. Emission spectra of the photoreactions of SDBS-(6,5)-SWCNTs with **NO_2ArNNS** formed *in situ.* **NO_2ArNNS** was formed in the SDBS-(6,5)-SWCNTs solution at various concentrations of **NO_2ArDz** and Na_2SO_3 combinations irradiated with 527 nm green LED light. a) **NO_2ArDz** added = 50 μM, Na_2SO_3 = 5 mM. b) **NO_2ArDz** added = 100 μM, Na_2SO_3 = 5 mM. c) **NO_2ArDz** added = 200 μM, Na_2SO_3 = 5 mM. d) **NO_2ArDz** added = 200 μM, Na_2SO_3 = 2.5 mM. e) **NO_2ArDz** added = 200 μM, Na_2SO_3 = 1 mM. f) Control measurement in the absence of Na_2SO_3 (**NO_2ArDz** added = 200 μM, Na_2SO_3 = 0 mM).

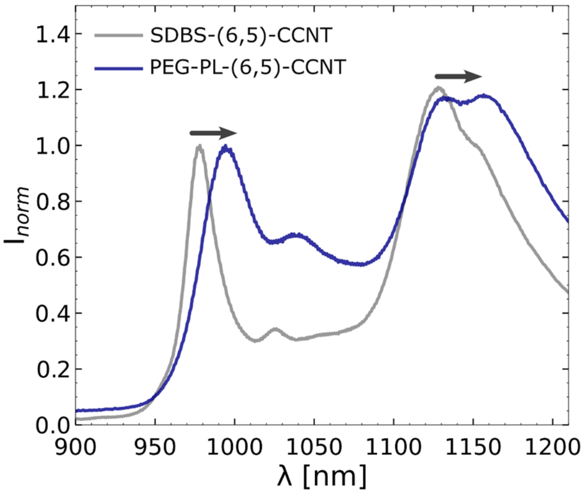

Figure S21. Surfactant exchange of (6,5)-CCNTs prepared by the photoreaction with **CoumNNS** from SDBS (grey) to PEG-PL (blue).

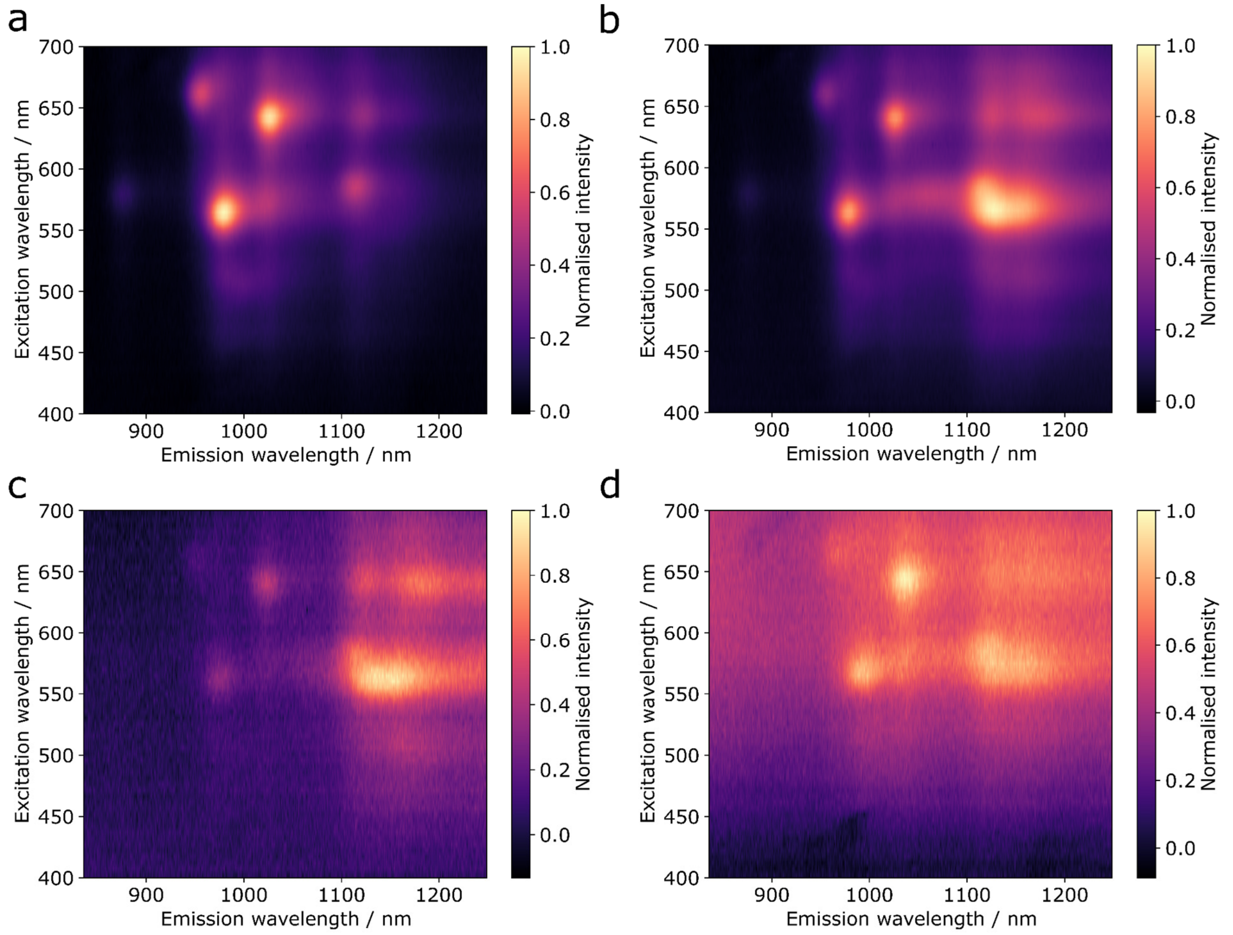

Figure S22. Normalized 2D spectra of a) Pristine SDBS-(6,5)-SWCNTs, b) **CoumNNS** (40 μM, see section 6.2.)- modified SDBS-(6,5)-CCNTs, and c) CoumNN (40 μM, see section 5.1.)- modified SDBS-(6,5)-CCNTs and d) PEG-PL-(6,5)-CCNTs.

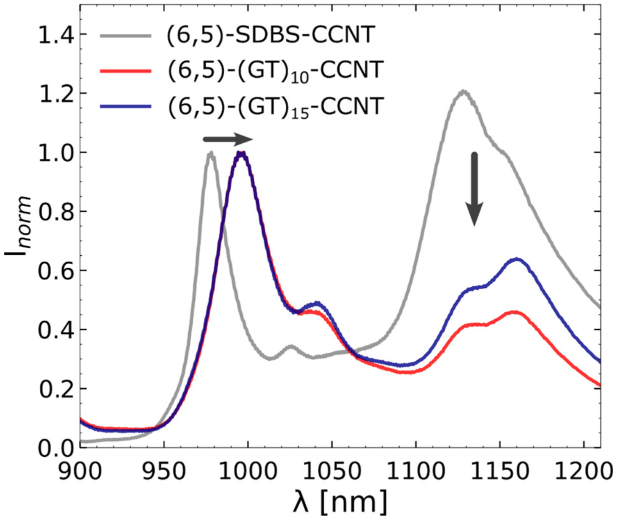

Figure S23. Surfactant exchange of (6,5)-CCNTs prepared by photoreaction with **CoumNNS** from SDBS to $(GT)_{10}$ (red) and $(GT)_{15}$ (blue).

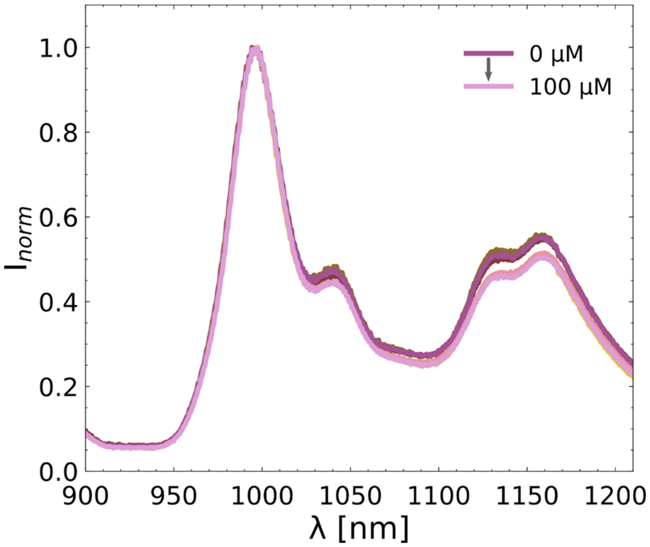

Figure S24. Emission spectra normalized to the E_{11} peak before and after the addition of 100 μM of dopamine to the (6,5)- $(GT)_{15}$ -CCNTs sample in PBS.

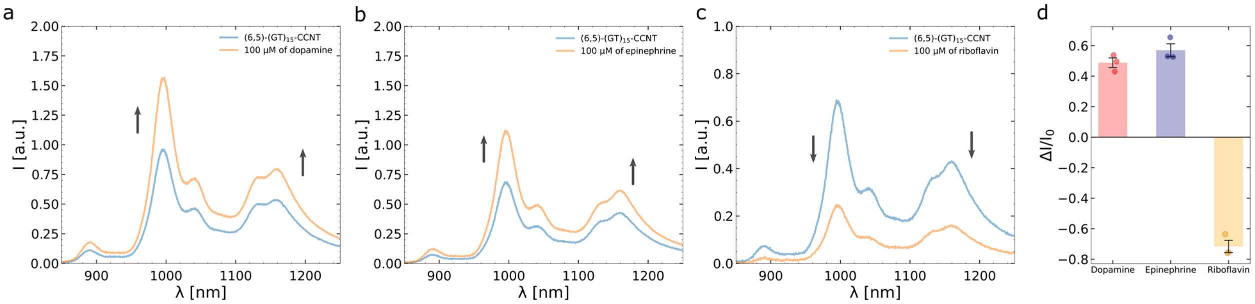

Figure S25. Emission intensity changes ($\Delta I/I_0$) after addition of 100 μM of a) dopamine, b) epinephrine, and c) riboflavin in PBS. d) Overall emission intensity changes ($\Delta I/I_0$) after addition of 100 μM of various analytes in PBS (mean ± SE, n=3).

References

[1] J. T. Metternich, J. A. C. Wartmann, L. Sistemich, R. Nißler, S. Herbertz, S. Kruss, "Near-Infrared Fluorescent Biosensors Based on Covalent DNA Anchors" *J. Am. Chem. Soc.* **2023**, *145*, 14776–14783.

[2] F. Schöppler, C. Mann, T. C. Hain, F. M. Neubauer, G. Privitera, F. Bonaccorso, D. Chu, A. C. Ferrari, T. Hertel, "Molar Extinction Coefficient of Single-Wall Carbon Nanotubes" *J. Phys. Chem. C* **2011**, *115*, 14682–14686.

[3] C. Ma, C. A. Schrage, J. Gretz, A. Akhtar, L. Sistemich, L. Schnitzler, H. Li, K. Tschulik, B. S. Flavel, S. Kruss, "Stochastic Formation of Quantum Defects in Carbon Nanotubes" *ACS Nano* **2023**, *17*, 15989–15998.

[4] F. A. Mann, P. Galonska, N. Herrmann, S. Kruss, "Quantum defects as versatile anchors for carbon nanotube functionalization" *Nat. Protoc.* **2022**, *17*, 727–747.

[5] N. Jiang, Q. Huang, J. Liu, N. Liang, Q. Li, Q. Li, S.-S. Xie, "Design, synthesis and biological evaluation of new coumarin-dithiocarbamate hybrids as multifunctional agents for the treatment of Alzheimer's disease" *European Journal of Medicinal Chemistry* **2018**, *146*, 287–298.

[6] M.-Z. Zhang, R.-R. Zhang, W.-Z. Yin, X. Yu, Y.-L. Zhang, P. Liu, Y.-C. Gu, W.-H. Zhang, "Microwave-assisted Synthesis and antifungal activity of coumarin[8,7-e][1,3]oxazine derivatives" *Mol Divers* **2016**, *20*, 611–618.

[7] J. K. Streit, J. A. Fagan, M. Zheng, "A Low Energy Route to DNA-Wrapped Carbon Nanotubes via Replacement of Bile Salt Surfactants" *Anal. Chem.* **2017**, *89*, 10496–10503.
